# GABA regulates metabolic reprogramming to mediate the development of brain metastasis in non-small cell lung cancer

**DOI:** 10.1186/s13046-025-03315-9

**Published:** 2025-02-19

**Authors:** Mengqing Xie, Hao Qin, Li Liu, Jing Wu, Zhikai Zhao, Yaodong Zhao, Yujia Fang, Xin Yu, Chunxia Su

**Affiliations:** 1 Department of Medical Oncology, Shanghai Pulmonary Hospital, Tongji University School of Medicine, Tongji University, Shanghai, China; 2 Department of Thoracic Surgery, Huadong Hospital, Fudan University, Shanghai, China; 3 Department of Pathology, Shanghai Pulmonary Hospital, Tongji University School of Medicine, Tongji University, Shanghai, China; 4 Department of Neurosurgery, First Affiliated Hospital of Shanghai Jiao Tong University, Shanghai Jiao Tong University, Shanghai, China

**Keywords:** GABA, NSCLC, Brain metastasis, Astrocyte, Metabolic reprogramming

## Abstract

**Background:**

Brain metastasis (BrM) poses a significant challenge to the prognosis and quality of life for patients with non-small cell lung cancer (NSCLC). Gamma-aminobutyric acid (GABA), an inhibitory neurotransmitter in the central nervous system (CNS), has been implicated in the progression of various tumors. However, its potential role in BrM of NSCLC and the underlying mechanisms remain largely unexplored.

**Methods:**

A multi-omics approach combined with in vivo and in vitro experiments identified GABA as a key target in BrM of NSCLC. Functional and mechanistic studies were conducted to investigate how GABA mediates brain metastasis through the activation of the NF-κB pathway.

**Results:**

GABA levels were significantly elevated in both cells and serum of patients with NSCLC who had BrM. GABA markedly enhanced the brain metastatic capabilities and malignancy of NSCLC cells. Mechanistically, tumor cells with a tendency for brain metastasis can inhibit 4-aminobutyrate aminotransferase (ABAT) by downregulating forkhead box A2 (FOXA2) expression, leading to increased GABA accumulation. GABA subsequently activates the NF-κB pathway and the astrocytes, thus facilitating the brain metastasis of NSCLC.

**Conclusions:**

Our findings indicate that GABA plays a crucial role in the development of NSCLC brain metastasis by activating the NF-κB pathway through the FOXA2/ABAT/GABA axis. Additionally, the interaction between NSCLC and astrocytes creates an inhibitory microenvironment that promotes tumor colonization.

**Supplementary Information:**

The online version contains supplementary material available at 10.1186/s13046-025-03315-9.

## Introduction

Lung cancer is a malignant tumor with the highest mortality rate worldwide [1]. Metastasis is the main cause of death in lung cancer patients, and the brain is one of the most common distant metastasis sites [2]. About 20% of patients already have brain metastases when they are first diagnosed, and 40-50% develop brain metastases during the disease [3]. Brain metastasis leads to neurological complications that significantly impact patients’ quality of life [4, 5]. In recent years, despite the breakthroughs in systemic treatment of NSCLC [6, 7], NSCLC with brain metastasis have limited treatment options. Therefore, we urgently need to improve our understanding of the underlying mechanisms with brain metastasis.

Cancer cells undergo significant metabolic reprogramming during metastasis to adapt to the microenvironment, with different metastatic sites presenting distinct metabolic challenges [8]. “Glutamine addiction” is an important part of the tumor metabolic reprogramming process [9], but its downstream non-protein amino acid GABA is less studied. As the predominant inhibitory neurotransmitter in the CNS, GABAergic signaling plays a vital role in the brain, functioning in signal transduction and homeostatic regulation [10, 11]. Beyond the CNS, GABA has also been found to promote the proliferation and progression of certain solid tumors through various mechanisms [12–14]. However, as a unique substance of the CNS, whether tumor cells could express GABA through metabolic reprogramming to promote brain metastasis has rarely been reported [15].

Astrocytes are the primary glial cells in the brain microenvironment and are in close contact with neurons. Previous studies have demonstrated that tumor cells can enhance their colonization of the brain by interacting with astrocytes, effectively recruiting these cells to support tumor growth [16, 17]. However, it remains unknown whether brain metastatic cells can simulate neuronal activity by secreting GABA to tame astrocytes and create a microenvironment conducive to the survival of tumor cells.

In this study, we conducted a comprehensive exploration of GABA in the brain metastasis of NSCLC. Our findings reveal the potential mechanism by which tumor cells promote brain metastasis by altering their metabolism to mimic the CNS microenvironment. We identified high levels of GABA and its role in enhancing brain metastasis in NSCLC, elucidating the underlying mechanism involving the FOXA2/ABAT/GABA-dependent metabolic pathway. Additionally, we discovered that astrocytes influenced by GABA also contribute to the promotion of metastasis. In summary, our study investigates the role and mechanisms of GABA in lung cancer brain metastasis from different perspectives, uncovering its biological function and its impact on brain metastasis.

## Methods

### Cell culture

The human NSCLC cell lines, H460, PC9, and 293T were originally purchased from Cell Bank of the Chinese Academy of Sciences (Shanghai, China). Lentiviral transfection was performed to establish the corresponding cell lines (H460-luciferase (Luc), H460-green fluorescent protein (GFP), PC9-Luc, PC9-GFP). Both cells were cultured in DMEM (Gibco, Grand Island, New York, USA) plus 10% fetal bovine serum (FBS, Gibco, Grand Island, New York, USA) with 1% penicillin/streptomycin (P/S, Gibco, Grand Island, New York, USA). H460BrM-luc and PC9BrM-luc were obtained through at least three cycles of left ventricular injection and cultured in vitro. All the BrM cells were used in the 10 generations. Human astrocyte HA1800 were purchased from Shanghai WHELAB Bioscience and cultured in DMEM. Primary mouse astrocytes (mA) were prepared from the cerebral cortex of 2-day-old mice and cultured in DMEM.

### Cell proliferation

Cell counting kit 8 (CCK 8): 5*10^3 cells were seeded in 96-well plates, and the CCK8 solution was added at different times (Biosharp, China). The optical density was measured at 450 nm using a spectrophotometer.

Colony formation assays: 5*10^2 cells were seeded in 6-well plates and culture for 14 days. Colonies were fixed with methanol for 15 min and stained by crystal violet solution for 30 min.

5-ethynyl-2′-deoxyuridine (EdU) incorporations: The EdU incorporation assay was performed using a commercial kit (CellorLab, Shanghai, China). Cells were inoculated in 24-well plates for 24 h, and incubated in EdU at a concentration of 10 µM for 2 h. Cells nuclei were stained with Hoechst 33,342. Fluorescence microscopy was performed to calculate the percentage of EdU-positive cells.

### Cell apoptosis

Cells after treatment were collected and washed with phosphate-buffered saline (PBS). The cells were then resuspended in binding buffer at a concentration of 1*10^6 cells/mL. Annexin V-FITC and propidium iodide were added according to the manufacturer’s instructions (BD Biosciences, Franklin Lakes, NJ, USA). After a 15-minute incubation in the dark at room temperature, the cells were analyzed using a flow cytometer (BD Biosciences).

### Wound healing assay

Cells were cultured in 6-well plate, after cells attached, a pipette tip was used to create an incision-like gap. Cell migration is quantified and expressed as average percentage of closure of the scratch area.

### Cell migration and invasion assays

The cell migration and invasion assay were performed using Transwell chambers (8 μm, Corning Inc.). 1*10^5 cells were seeded in the upper chamber and treated with serum-free medium. The lower chamber contained complete medium with 10% FBS. As for the invasion assay, the bottom membrane of the upper chamber was coated with Matrigel (1:10 dilution, BD Biosciences) and incubated at 37 °C for 30 min to allow for gelation.

The cells that penetrated through the membrane to the lower surface were fixed with methanol and stained with crystal violet for 15 min. Images were captured under a microscope, and the number of migratory cells in randomly selected fields was counted.

### Trans-endothelial assays

hCMEC/D3 cells (1*10^5) were seeded into the upper wells of a Transwell chamber and cultured until a confluent monolayer was formed. Tumor cells (5 *10^4) suspended in medium containing 1% FBS were then added to the upper inserts, while 500 µl of medium supplemented with 20% FBS was placed in the lower chamber. After 24 h, GFP-labeled tumor cells that had migrated through the membrane were visualized and quantified using a fluorescent microscope.

### Inflammasome activation

LPS and ATP were utilized to activate the NLRP3 inflammasome, MCC950 served as an inhibitor of NLRP3 inflammasome activation. HA1800 cells were initially exposed to LPS (1 µg/mL) for 6 h. Following this, the cells were pretreated with serum-free medium containing either DMSO (1:1000) or MCC950 (10 µM) for 1 h, before being stimulated with ATP (5 mM) for an additional hour.

### Cell transfection

The ABAT overexpressing lentivirus and the control lentivirus were bought from OBIO (Shanghai, China). The H460BrM and PC9BrM cells were lentiviral transduced and selected with 5 µg/ml puromycin for 5 days to get ABAT overexpression (OE) cells.

The siRNAs against FOXA2 were purchased from OBIO (Shanghai, China). H460 and PC9 cells were transfected with siRNA using the Lipofectamine 2000 (Invitrogen) according to the manufacturer’s instructions. The cells were harvested at 48 h after transfection. A non-specific scramble siRNA sequence was used as negative control (NC). The sequences of siRNAs are summarized in Supplementary Table S1.

### RNA sequencing and metabolome sequencing

Differently treated cells were sent to Majorbio Inc. (Shanghai. China) for RNA and metabolome sequencing. In total, messenger RNA was extracted according to instructions, metabolites were analyzed using liquid chromatography coupled-mass spectrometry (LC-MS). The resulting data were processed using bioinformatics tools for quality control, alignment to the reference genome, and differential expression analysis and subsequent analyses.

### Animal experiments

For subcutaneous experiments, 1*10^6 cells were injected into flank of 6 weeks old BALB/c nude mice (GemPharmatech Co., Ltd). Tumor sizes were measured every 4 days. Tumor weight was recorded, and volumes were estimated according to 1/2 × (length × width^2^).

For left ventricular injection experiments, 1*10^5 cells were injected into left ventricular of 6 weeks old BALB/c nude mice (GemPharmatech Co., Ltd). Brain metastasis was confirmed via in vivo bioluminescence imaging and hematoxylin-eosin (HE) staining approximately 30 days following left ventricular injection or upon the observation of clinical signs such as weight loss, reduced mobility, or neurological symptoms in the mice. Specifically, mice received an intraperitoneal injection of 150 µL of D-luciferin (Synchem, YG100232) and were anesthetized with 1.5% sodium pentobarbital. Imaging was performed using a Bioluminescence Imaging System (Tanon 6600, China; Tanon ABL X5 Pro, China). Brain metastasis was detected, and bioluminescence imaging was quantified and analyzed in terms of photons per pixel (PPP) or radiance (p/sec/cm^2^/sr). Subsequently, all mice were euthanized, and their brains were excised. Histological analysis was conducted using HE staining, with sections prepared at 10 mm intervals and stained accordingly.

GABA was administered intraperitoneally five days after the left ventricle injection. The mice were randomly divided into an experimental group and a control group. The experimental group received GABA (40 mg/kg) dissolved in 200 µL of PBS, while the control group was given 200 µL of PBS. Both groups were treated three times a week for a duration of three weeks.

The institutional guidelines for animal care and use were strictly followed, and all procedures were approved by the Institutional Review Board of Shanghai Pulmonary Hospital.

### Clinical sample collection

Clinical data and samples from patients with NSCLC were collected from Shanghai Pulmonary Hospital. Blood and formalin-fixed paraffin-embedded (FFPE) samples were obtained at baseline for research purposes, with approval from the Ethical Review Committee. All procedures were conducted in accordance with the Declaration of Helsinki. Blood samples were centrifuged at 12,000 rcf for 10 min within 2 h of collection to obtain plasma, which was then stored at -80 °C. FFPE samples were also obtained for immunohistochemistry (IHC).

### Quantitative real-time polymerase chain reaction (qPCR)

Total RNA was extracted by RNA extraction kit (AGbio, Shanghai, China). cDNA was synthesized using a cDNA Synthesis Kit (AGbio, Shanghai, China). qPCR was performed according to manufacturer’s protocol (Qiagen 208056). The Ct values were acquired and contrasted using the 2 − ΔΔCt method and values were normalized against β-actin. The primers sequences used were listed in Supplementary Table S1.

### Western blot (WB)

Cell lysates were prepared in RIPA lysis buffer containing 1% protease inhibitor cocktail (Beyotime). After electrophoresed on 7.5–12.5% SDS-PAGE gels, proteins were transferred to PVDF membranes (Millipore), and blocked with 5% skim milk for 1 h. β-actin antibody served as the internal control.

Primary antibodies were as follows: β-actin (Cell Signaling Technology, 4967); ABAT (Proteintech, 11349-1-AP); GAD1 (Proteintech, 10408-1-AP); GAD2 (Proteintech, 20746-1-AP); NF-κB p65 (Cell Signaling Technology, D14E12); phospho-NF-κB p65 (Cell Signaling Technology, 3033); phospho-IκBα (Cell Signaling Technology, 2859); FOXA2 (Proteintech, 22474-1-AP); GATA2 (SantaCruz, sc-267); CREB-1 (SantaCruz, sc-271), and incubated at 4℃ overnight. The corresponding horseradish peroxidase-conjugated secondary antibody was then applied for 1 h at room temperature before chemiluminescence detection.

### Enzyme linked immunosorbent assay (ELISA)

Plasm, cell lysates and cell supernatant were collected and used for the detection of GABA by human cytokines ELISA (Lengton, Shanghai, China) kits according to the manufacturer’s instructions.

### Adenosine triphosphate (ATP) measurement

Cell lysates after different treatment were collected, a luminescent ATP detection assay kit (Beyotime, Haimen, China) was employed to detect the ATP level according to the manual.

### Dual-luciferase reporter assay

After seeding in a 24-well plate, 293T cells were co-transfected with either the ABAT promoter-reporter gene vector or its mutant, along with the Renilla luciferase vector and either FOXA2 pcDNA or pcDNA3.1, using Lipofectamine 2000 (Invitrogen). Following 48 h of transfection, cell lysates were prepared using the Dual-Luciferase Reporter Assay System (Beyotime, Haimen, China). Renilla luciferase activity served as the internal control for normalizing luciferase activity.

### Immunohistochemical (IHC) and multiplex immunofluorescence assays (mIF)

For IHC, tissue sections were blocked with 5% bovine serum albumin in PBS. Primary antibodies were applied to the sections and incubated overnight at 4 °C. Then, sections were incubated with secondary antibodies for 1 h at room temperature. Visualization was achieved using a chromogenic substrate and sections were counterstained with hematoxylin. Images were captured using a light microscope.

For mIF, multiple primary antibodies targeting different proteins were diluted and applied simultaneously to the sections, followed by incubation overnight at 4 °C. Then, sections were incubated with fluorescently labeled secondary antibodies for 1 h at room temperature. Nuclei were counterstained with DAPI. Images were captured using a fluorescence microscope, and the fluorescence intensities of the respective proteins were quantified using appropriate image analysis software.

The antibodies employed for IHC and mIF were as follows: Ki-67 (Servicebio, GB111141), cleaved-caspase-3 (Servicebio, GB11532), GABA (Sigma-Aldrich, A2052), ABAT (Proteintech, 11349-1-AP), IBA-1 (Servicebio, GB15105) and GFAP (Servicebio, GB12100).

### Statistical analysis

Statistical analyses were conducted using SPSS version 23.0 (SPSS Inc., Chicago, IL, USA). Categorical variables were analyzed using the Chi-square test or Fisher’s exact test, as appropriate. Continuous variables were compared using the Mann-Whitney U test, Student’s t-test, or one-way ANOVA, depending on the distribution of the data and the number of groups being compared. The Kaplan-Meier method was employed to estimate overall survival (OS). A two-sided p-value of < 0.05 was considered statistically significant.

## Results

### Comprehensive multi-omics analysis of NSCLC with brain metastasis

**Fig. 1:**
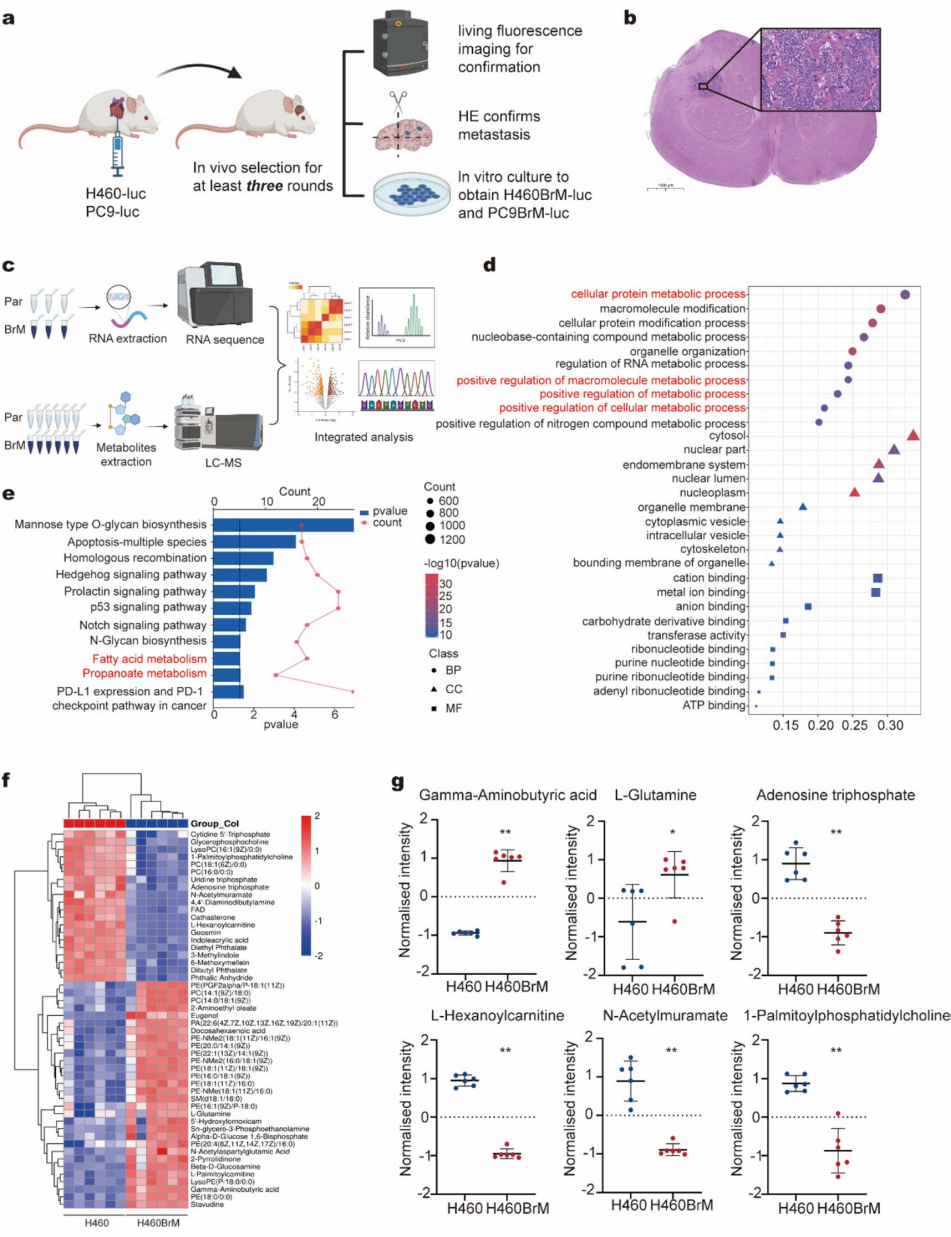
Identify GABA as a key factor for brain metastasis of NSCLC using multi-omics analysis (**a**) Schematic representation of the in vivo selection and confirmation of NSCLC cells with brain metastasis. (**b**) Representative image of hematoxylin-eosin staining demonstrating NSCLC with brain metastasis. (**c**) Schematic diagram illustrating the workflow for transcriptome and metabolome analysis. (**d**) Gene Ontology enrichment analysis comparing H460 and H460BrM. (**e**) Kyoto Encyclopedia of Genes and Genomes enrichment analysis between H460 and H460BrM. (**f**) Heat map analysis of metabolite compositions in H460 versus H460BrM. (**g**) Quantification of gamma-aminobutyric acid (GABA), L-glutamine, adenosine triphosphate, L-hexanoylcarnitine, N-acetylmuramate, and 1-palmitoylphosphatidylcholine levels in H460 and H460BrM. *p < 0.05; **p < 0.01. Par, Parental cells; BrM, Brain metastasis cells. NSCLC, non-small cell lung cancer

To investigate the mechanisms underlying brain metastasis in NSCLC, we established brain metastasis models by injecting lung cancer cells into the left ventricle in a minimum of three rounds. (Fig. 1a). These cancer cells colonized the brains of BALB/c nude mice, forming large metastases, which were confirmed by in vivo imaging and HE staining (Fig. 1b, Fig. S1a-b). Compared with subcutaneous tumors and lung metastases, brain lesions displayed higher proliferation and apoptosis rates (Fig. S1c).

To identify the biological differences between parental and brain-metastatic cells, we isolated NSCLC cell subpopulations that preferentially metastasize to the brain, namely H460BrM and PC9BrM, through in vivo selection followed by in vitro culture. CCK 8 assays, EdU experiments, and colony formation assays indicated that brain-metastatic cells (H460BrM and PC9BrM) exhibited higher proliferation rates (Fig. S1d-f). We also assessed subcutaneous tumor formation in a mouse xenograft model, showing that H460BrM cells exhibited enhanced proliferative capacity. Both tumor volume and weight were significantly greater in the H460BrM group compared to the H460 group (Fig. S1g-i). Subsequently, transwell assays further demonstrated enhanced migratory and invasive capabilities in brain-metastatic cells compared to parental cells (Fig. S2a). Bioluminescence imaging revealed that H460BrM cells have a greater propensity for brain metastasis than parental cells, with the incidence of brain metastases increasing from 16.7 to 66.7%, consistent with ongoing characterization (Fig. S2b-d). These findings suggest that brain-metastatic lung cancer cells possess a higher degree of malignancy.

Then, RNA sequencing and LC-MS untargeted metabolomics were conducted to compare parental and brain-metastatic cells, followed by multi-omics analysis (Fig. 1c). Gene Ontology and Kyoto Encyclopedia of Genes and Genomes enrichment analysis revealed that significantly differentially expressed genes were enriched in the metabolic process (Fig. 1d-e). Untargeted metabolomics provided a comprehensive overview of cancer metabolism in parental versus brain-metastatic lung cancer cells (Fig. 1f). In the brain-metastatic lung cancer cell H460BrM, levels of GABA and L-glutamine were significantly increased compared to H460, while metabolites such as ATP, L-hexanoylcarnitine, N-acetylmuramate, and 1-palmitoylphosphatidylcholine were markedly reduced (Fig. 1g).

### Identify GABA as a key factor for brain metastasis of NSCLC both in vitro and in vivo

**Fig. 2:**
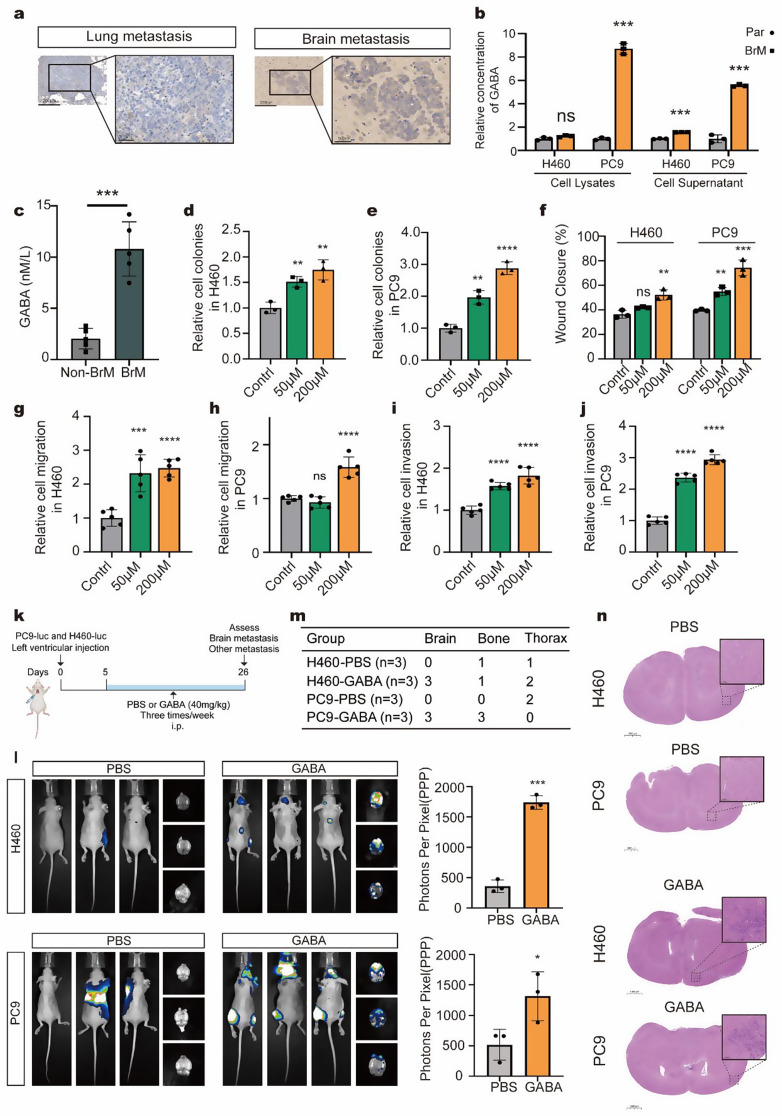
GABA promote the brain metastasis of NSCLC both in vitro and in vivo (**a**) Representative images of immunohistochemical staining showing GABA expression in lung versus brain metastases. (**b**) Comparison of GABA concentrations in lysates and supernatants of parental (H460 and PC9) versus brain-metastatic cells (H460BrM and PC9BrM) using ELISA. (**c**) Comparison of serum GABA concentrations in NSCLC patients with and without brain metastasis. (**d**-**e**) H460 and PC9 cells were treated with 0, 50, and 200 µM GABA for 48 h to assess clonogenicity. (**f**) Wound healing assays were conducted to compare the migration capabilities of H460 and PC9 cells treated with different concentrations of GABA (0 µM, 50 µM, and 200 µM) for 48 h. (**g**-**j**) Transwell assays were conducted to compare the migration and invasion capabilities of H460 and PC9 cells treated with different concentrations of GABA (0 µM, 50 µM, and 200 µM) for 48 h. (**k**) Schematic representation of GABA treatment in mice. (**l**) Bioluminescence imaging of mice treated with PBS (200 µL) or GABA (40 mg/kg, 200 µL) following left ventricular injection of H460 and PC9 cells, along with a comparison of Photons Per Pixel between the two groups (*n* = 3). (**m**) The table summarized the metastasis status at different sites. (**n**) Representative brain images from hematoxylin-eosin staining in the PBS and GABA groups injected with H460 and PC9 cells. *p < 0.05; **p < 0.01; ***p < 0.001; ****p < 0.0001; ns, not significant

GABA has long been recognized as an inhibitory neurotransmitter in the CNS, and its accumulation in brain-metastatic cells piqued our interest. We assessed GABA expression in various lesions, finding that brain metastasis exhibited higher GABA levels compared to lung metastasis (Fig. 2a). To confirm these findings, we measured GABA concentrations in cell lysates and supernatants from parental and brain-metastatic cells. The results indicated that GABA was upregulated in both the supernatant and cell lysate of brain-metastatic cells (Fig. 2b). Furthermore, the level of GABA in advanced NSCLC patients with and without brain metastasis was investigated. The results revealed that those with brain metastasis had significantly higher serum GABA concentrations (Fig. 2c). Taken together, these findings suggest that GABA may play a crucial role in the brain metastasis of NSCLC.

To further evaluate the effects of GABA on the malignancy of NSCLC, H460 and PC9 cells were treated with various GABA concentrations (0 µM, 50 µM, 200 µM) for 48 h. We conducted colony formation assays, scratch assays, and, apoptosis assays. The results indicated that GABA enhanced proliferation (Fig. 2d-e, Fig. S3a-b) and wound closure (Fig. 2f, Fig. S3c-d) in NSCLC cells in a dose-dependent manner, without significantly affecting apoptosis (Fig. S3e-f). Subsequently, migration and transwell invasion experiments were performed to assess the migratory and invasive capabilities of the cells, revealing that GABA significantly promoted both migration (Fig. 2g-h, Fig. S3g) and invasion (Fig. 2i-j, Fig. S3h) in NSCLC. The ability of parental cells, brain metastatic cells, and GABA-treated parental cells to cross the blood-brain barrier (BBB) was also compared. The findings indicated that both brain metastatic cells and GABA-treated parental cells demonstrated an increased ability to cross the BBB compared to parental cells, though the differences were not significant (Fig. S3i-j).

Next, we examined the role of GABA in tumor brain metastasis in vivo using left ventricular cardiac injection (Fig. 2k). Bioluminescence imaging and HE staining confirmed that the GABA-treated group exhibited a greater propensity for brain metastasis compared to the control group (Fig. 2l-n). Overall, these findings demonstrate that GABA promotes the progression of NSCLC and its metastasis to the brain.

### GABA facilitate progression of NSCLC by activating the NF-κB pathway

**Fig. 3:**
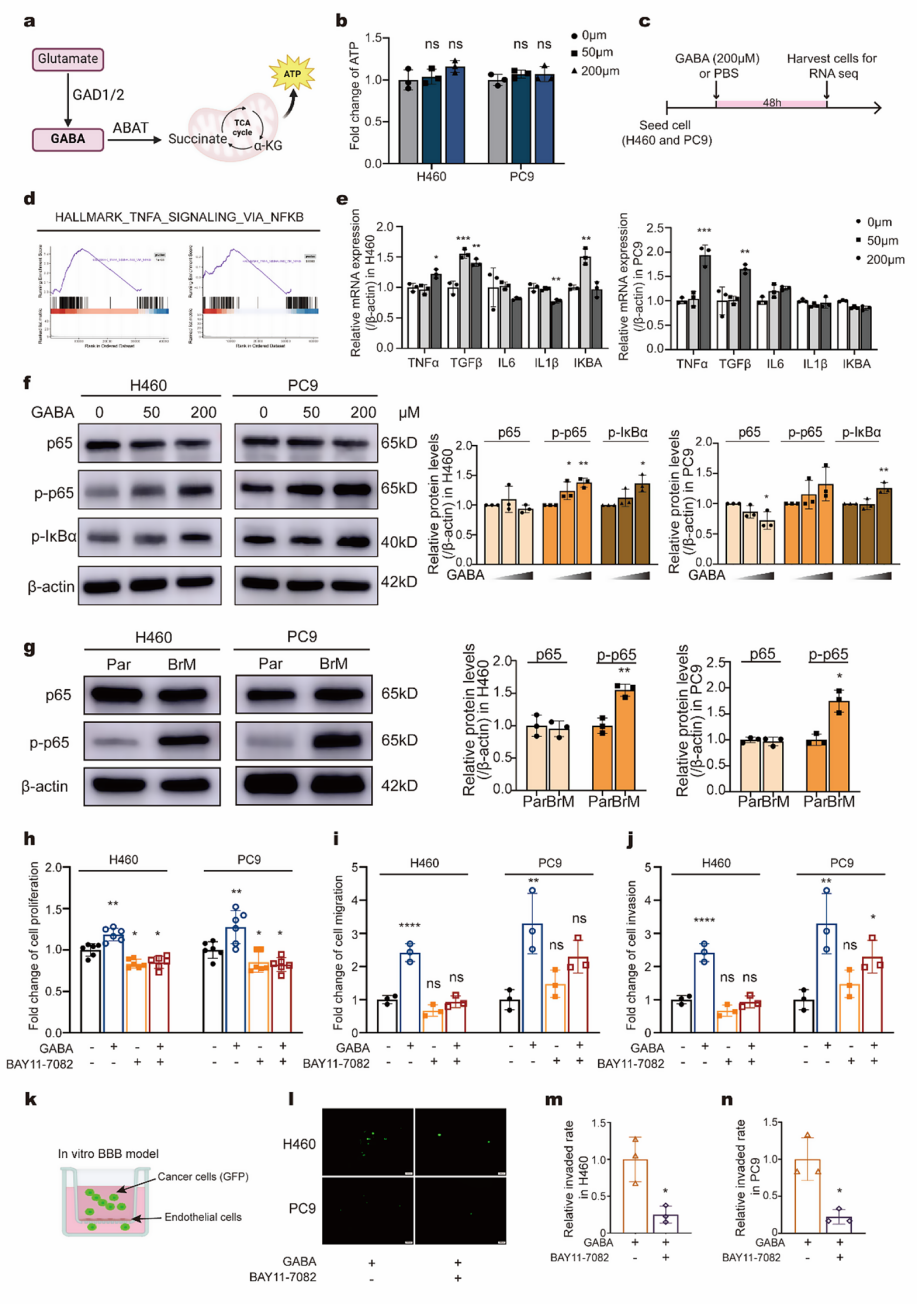
GABA facilitate brain metastasis of NSCLC by activating the NF-κB pathway (**a**) Schematic representation of intracellular glutamine metabolic flux in cells. (**b**) Fold change in adenosine triphosphate (ATP) levels in H460 and PC9 cells after treatment with varying concentrations of GABA (0 µM, 50 µM, 200 µM) for 48 h. (**c**) Schematic diagram outlining the workflow for RNA sequencing. (**d**) Gene set enrichment analysis of significant differential pathways between parental cells (H460 and PC9) and GABA-treated cells. (**e**) Changes in NF-κB pathway-related gene expression in H460 and PC9 cells following GABA treatment. (**f**) Western blot analysis showing the expression levels of p65, phosphorylated p65 (p-p65), and phosphorylated IκBα (p-IκBα) in H460 and PC9 cells treated with different concentrations of GABA (0 µM, 50 µM, 200 µM) for 48 h. (**g**) Western blot comparison of NF-κB signaling pathway between parental cells and brain metastatic cells. (**h**) Changes in proliferation of H460 and PC9 cells after treatment with GABA (200 µM) and BAY11-7082 (10 µM) for 48 h. (**i**) Changes in migration of H460 and PC9 cells after treatment with GABA (200 µM) and BAY11-7082 (10 µM) for 48 h. (**j**) Changes in invasion of H460 and PC9 cells after treatment with GABA (200 µM) and BAY11-7082 (10 µM) for 48 h. (**k**) Schematic diagram of the in vitro BBB model. (**l**-**n**) Comparison of the ability of GABA-treated cells to cross the BBB after BAY11-7082 treatment. *p < 0.05; **p < 0.01; ***p < 0.001; ****p < 0.0001; ns, not significant

Since GABA can provide energy by entering the tricarboxylic acid cycle (TCA) cycle or activate downstream pathways [18], we next explored its potential mechanisms in promoting NSCLC progression (Fig. 3a). We measured ATP levels in NSCLC cells treated with GABA to assess TCA cycle activity. The results indicated that treatment with various concentrations of GABA had a negligible effect on ATP levels (Fig. 3b), suggesting that exogenous GABA alone may not provide sufficient energy.

We then conducted RNA sequencing on H460 and PC9 to investigate the mechanisms by which GABA promotes brain metastasis in NSCLC (Fig. 3c). Gene set enrichment analysis of all detected upregulated and downregulated genes revealed activation of the NF-κB signaling pathway as a point of interest (Fig. 3d). Previous studies have indicated that the NF-κB pathway plays a crucial role in various biological processes, including cell proliferation, growth, and tumor progression [19, 20].

To further explore this, we performed WB and qPCR to assess changes in genes involved in the NF-κB pathway in NSCLC. Most NF-κB-related genes, including TNFα, TGFβ, and IL6 were found to be upregulated by GABA (Fig. 3e). WB analysis further demonstrated that GABA increased the phosphorylation of p65 and IκBα in a dose-dependent manner, with a difference observed at 200 µM, indicating activation of the NF-κB signaling pathway (Fig. 3f). Additionally, we compared the expression of NF-κB signaling pathway between brain metastatic cells and parental cells. Consistent with our hypothesis, brain metastatic cells exhibited activation of the NF-κB signaling pathway (Fig. 3g, Fig. S4a-b). This finding underscores the inherent differences in NF-κB signaling between brain metastatic and parental cell lines, providing important insights into the mechanisms that may contribute to brain metastasis.

Recognizing the importance of GABA-mediated NF-κB activation, we further examined whether inhibiting this pathway could mitigate GABA-induced NSCLC progression. The results showed that inactivation of the NF-κB pathway using BAY11-7082 consistently reduced GABA-mediated proliferation (Fig. 3h), migration (Fig. 3i), and invasion (Fig. 3j) in H460 and PC9. Furthermore, BAY11-7082 treatment reduced the ability of GABA-treated cells to cross the BBB (Fig. 3k-n). Together, our data indicate that GABA facilitates the progression of NSCLC by activating the NF-κB pathway.

### Loss of ABAT during brain metastasis increase the GABA and promote the malignancy of NSCLC

**Fig. 4:**
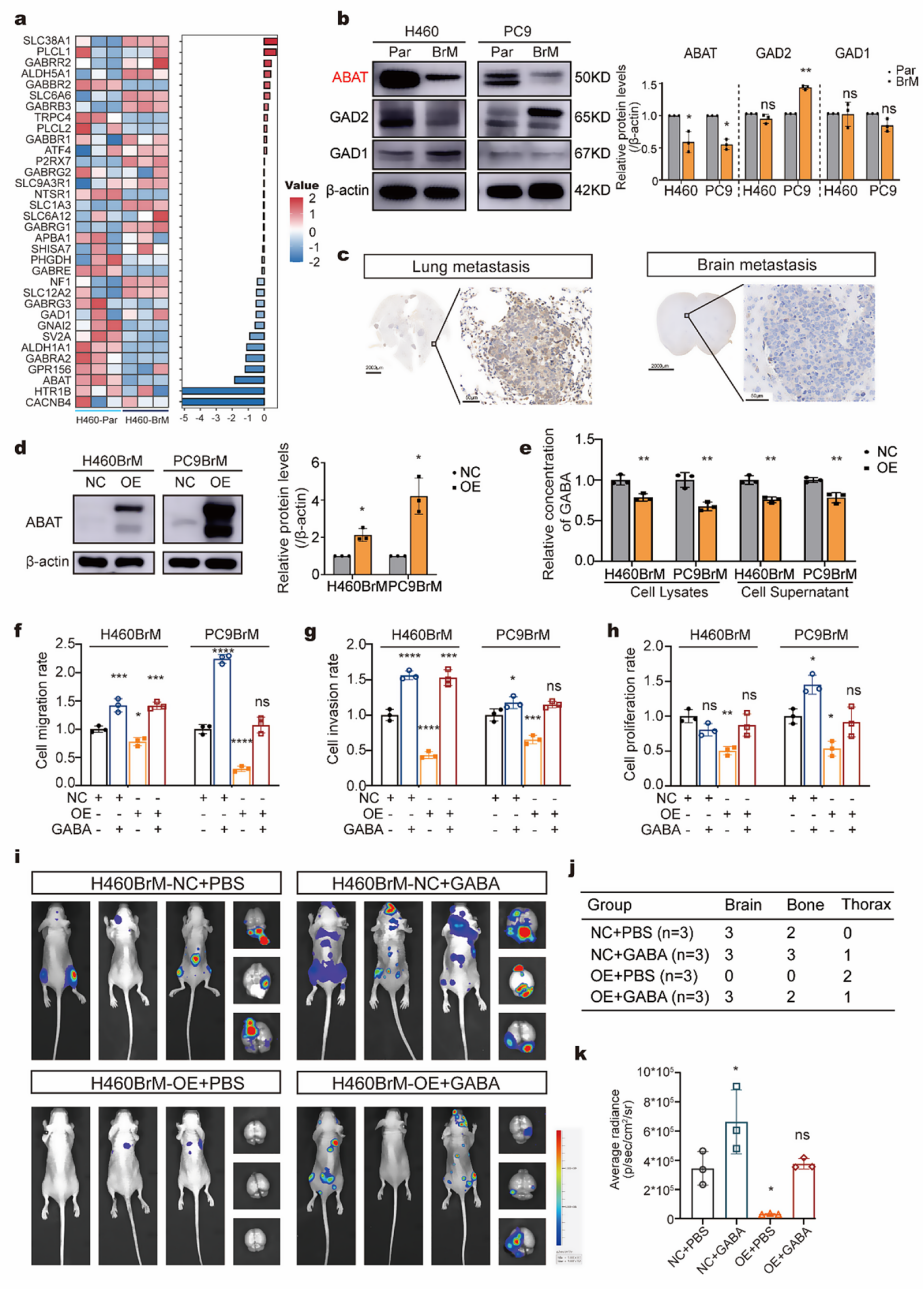
Loss of ABAT during brain metastasis increase the GABA and promote the malignancy of NSCLC (**a**) Heat map illustrating changes in the expression of genes involved in GABA between H460 and H460BrM cells. (**b**) Western blot analysis of GABA-related protein (GAD1, GAD2, and ABAT) expression in brain-metastatic cells (H460BrM and PC9BrM) versus parental (H460 and PC9). (**c**) Immunohistochemical staining demonstrating ABAT expression in lung metastases versus brain metastases. (**d**) Confirmation of ABAT overexpression at the protein level in H460BrM and PC9BrM cells. (**e**) Comparison of GABA concentrations in cell lysates and supernatants between control cells (H460BrM-NC and PC9BrM-NC) and overexpressing cells (H460BrM-OE and PC9BrM-OE) using ELISA. (**f**-**h**) Differences in (**f**) migration, (**g**) invasion, and (**h**) proliferation rates of brain-metastatic cells (H460BrM and PC9BrM) in the NC and OE groups following exogenous GABA treatment (200 µM). (**i**) Bioluminescence imaging of mice after left ventricular injection of H460BrM-NC and H460BrM-OE cells, supplemented with exogenous GABA (*n* = 3). (**j**) The table summarized the metastasis status at different sites. (**k**) Comparison of brain radiance signals among different groups. *p < 0.05; **p < 0.01; ***p < 0.001; ns, not significant. NC, negative control; OE, overexpression. GAD1, Glutamate decarboxylase 1; GAD2, Glutamate decarboxylase 2; ABAT, 4-aminobutyrate aminotransferase

To gain insight into the mechanism underlying increased GABA levels, we analyzed RNA sequencing results from parental and brain metastatic cells, highlighting the differentially expressed genes involved in GABAergic properties (Fig. 4a). We performed qPCR to verify the expression of genes related to the synthesis, catabolism, and transport of GABA. The qPCR results indicated that ABAT, responsible for GABA catabolism, was significantly downregulated in both H460BrM and PC9BrM cells compared to parental cells (Fig. S5a). Further comparison of glutamate decarboxylase 1 (GAD1), glutamate decarboxylase 2 (GAD2), and ABAT expression between parental and brain metastatic cells confirmed the downregulation of ABAT in brain metastatic cells (Fig. 4b). IHC analysis of ABAT in brain metastases and lung metastases also showed lower expression levels in brain metastases (Fig. 4c). These findings underscore the critical role of ABAT in the process of NSCLC brain metastasis.

We subsequently overexpressed ABAT in H460BrM and PC9BrM cells (Fig. 4d, Fig. S5b). Overexpression of ABAT led to decreased GABA production in both cell lines (Fig. 4e). The overexpression of ABAT significantly reduced the migration, invasion, and proliferation. These inhibitory effects were partially reversed by the administration of exogenous GABA (200 µM) (Fig. 4f-h). To further confirm these findings, we investigated the role of ABAT in NSCLC in vivo, revealing that high ABAT expression levels reduced the brain metastasis, tumor weight and volume (Fig. 4i-k, Fig. S5c-e). Collectively, these results suggest that loss of ABAT promotes brain metastasis and tumor growth in NSCLC by increasing GABA levels.

### FOXA2 activates ABAT transcription

**Fig. 5:**
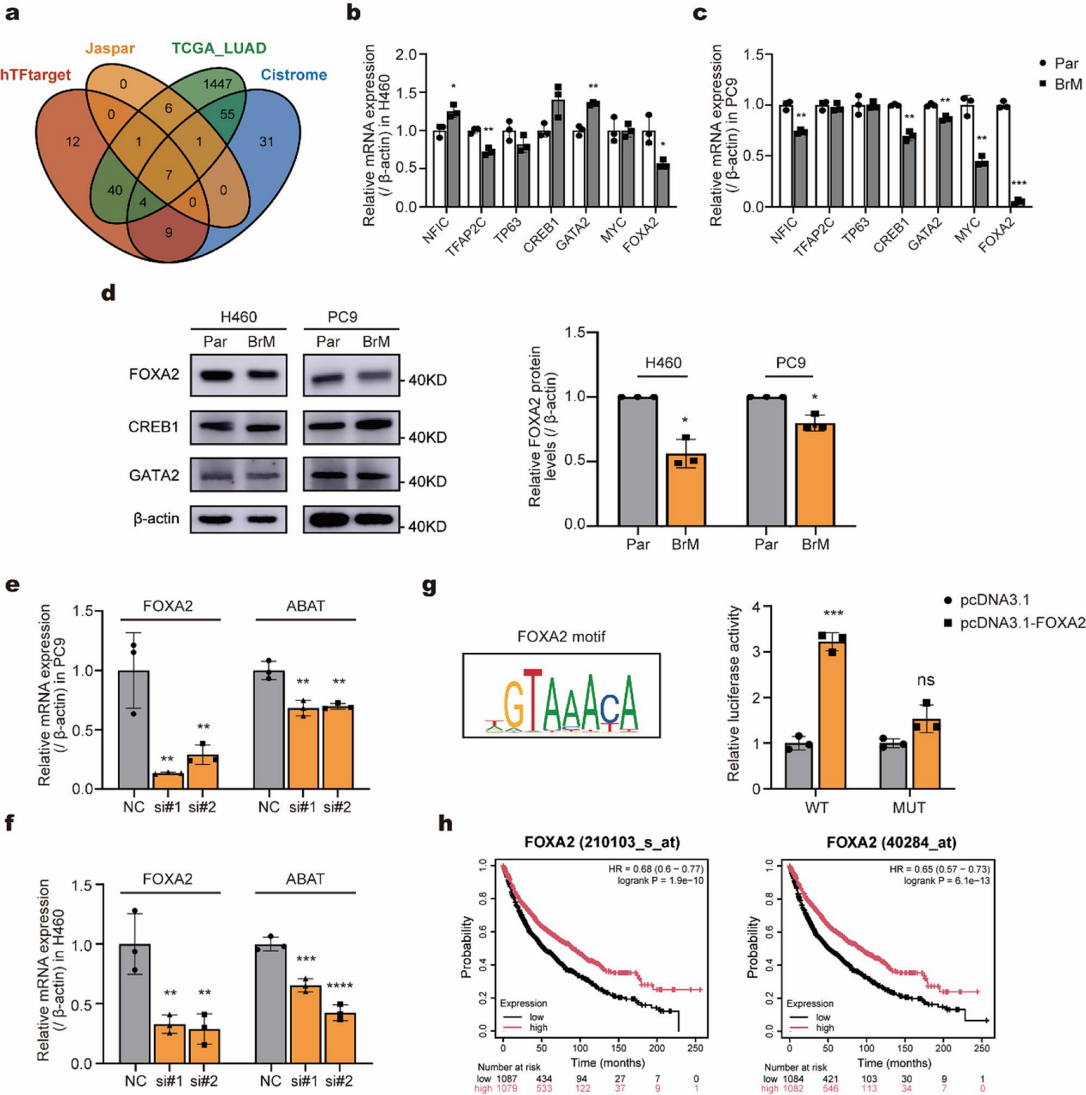
FOXA2 activates ABAT transcription (**a**) Prediction of potential upstream transcription factors of ABAT using a database. (**b**-**c**) Changes in transcription factor expression in brain-metastatic cells (H460BrM and PC9BrM) versus parental (H460 and PC9) assessed by qPCR. (**d**) Western blot analysis of transcription factor expression in brain-metastatic cells (H460BrM and PC9BrM) versus parental (H460 and PC9). (**e**-**f**) Effect of FOXA2 knockdown on ABAT expression levels in PC9 and H460 cells, as determined by qPCR. (**g**) Luciferase activity in 293T cells with and without ectopic expression of FOXA2. (**h**) Overall survival curves stratified by FOXA2 expression levels. *p < 0.05; **p < 0.01; ***p < 0.001; ns, not significant

To investigate the regulation of ABAT transcription activation, we explored upstream transcription factors using online prediction tools (Fig. 5a). Molecular validation revealed that FOXA2 was the only factor exhibiting consistent trends across both groups. The expression of FOXA2 was significantly decreased in H460BrM and PC9BrM cells compared to H460 and PC9 cells (Fig. 5b-d). Knockdown of FOXA2 resulted in substantial reductions in ABAT levels in both H460 and PC9 cells (Fig. 5e-f). Additionally, we elucidated the relationship between ABAT and FOXA2 using a dual luciferase reporter assay. The results showed that the mutated group exhibited significantly reduced luciferase activity compared to the wild-type group (Fig. 5g). And the CCK-8 assays and transwell assays showed that knokdown of FOXA2 increased the malignancy of brain metastatic cells overexpressing ABAT, supporting our hypothesis (Fig. S6a-b). We also examined the prognostic implications of FOXA2, low levels of FOXA2 were associated with poorer overall survival in NSCLC (Fig. 5h).

### Astrocytes are attracted to BrM cell-derived GABA and promote tumor progression through positive feedback

**Fig. 6:**
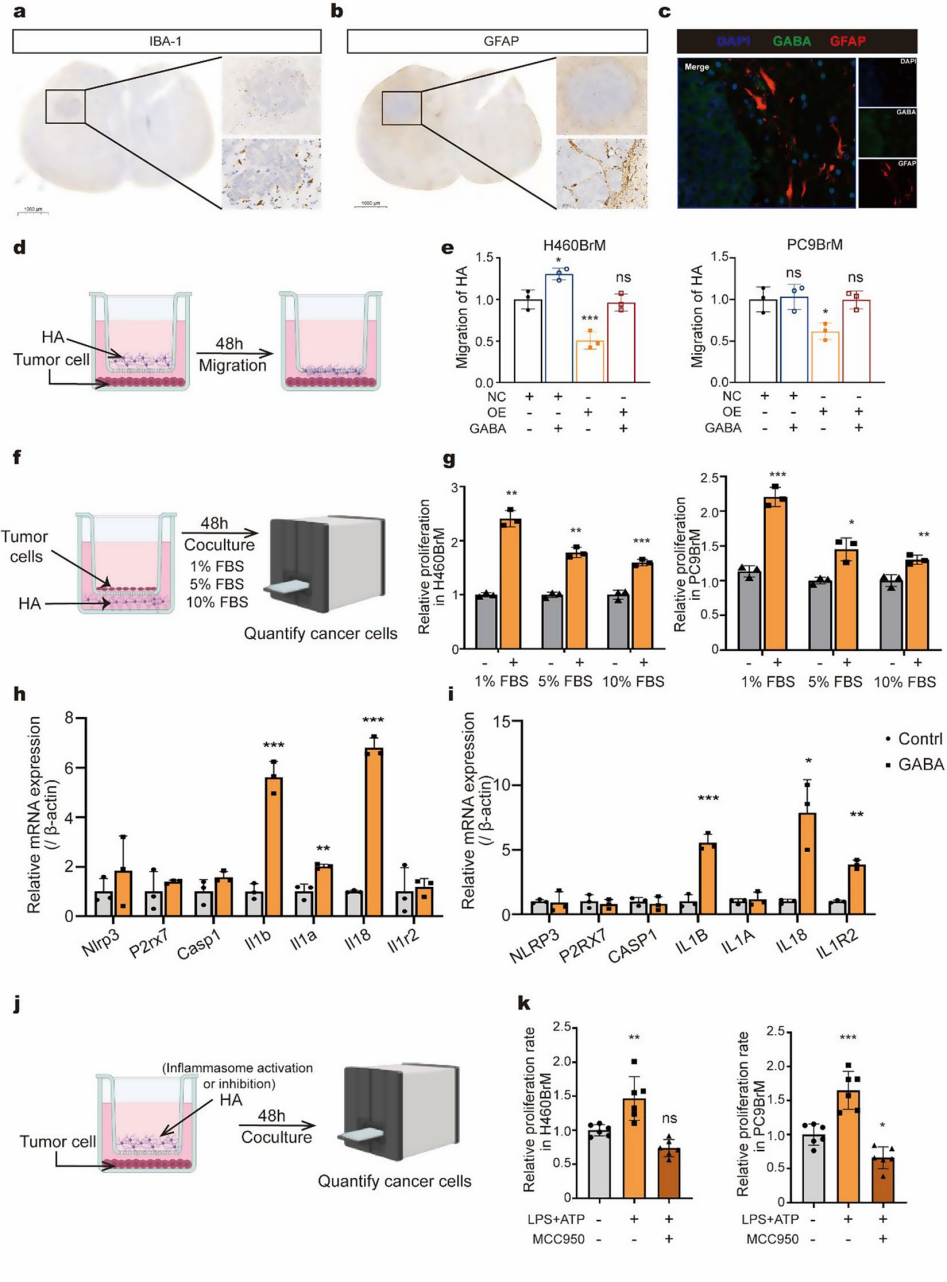
Astrocytes are attracted to BrM cell-derived GABA and promote tumor progression through positive feedback (**a**-**b**) Immunohistochemical staining for the expression of IBA-1 (microglia) and GFAP (astrocytes) in brain metastases. (**c**) Representative images of immunofluorescence staining for GABA and GFAP. (**d**-**e**) Co-culture of human astrocytes and tumor cells using transwell chambers, comparing the ability of tumor cells in the NC group and OE group to attract astrocytes after 48 h. (**f**-**g**) Co-culture of human astrocytes and tumor cells in transwell chambers to evaluate the effects of astrocytes on tumor cell proliferation at different serum concentrations (1% FBS, 5% FBS, 10% FBS). (**h**-**i**) Changes in NLRP3-related gene expression in (h) mouse astrocytes and (i) human astrocytes treated with different concentrations of GABA (0 µM and 200 µM), assessed by qPCR. (**j**-**k**) schematic diagram and comparison of the effects of NLRP3 activation or inhibition in human astrocytes on tumor cell proliferation. *p < 0.05, **p < 0.01, ***p < 0.001, ns: not significant. NC, negative control; OE, overexpression

The tumor microenvironment (TME) is known to influence both tumor progression and metastasis [21]. To explore the impact of the TME, we conducted IHC on brain metastasis. The results revealed that astrocytes (GFAP+) accumulated in the adjacent tissue and formed a barrier at the interface with the metastatic tissue, while microglia (IBA1+) did not exhibit similar accumulation (Fig. 6a-b). This finding piqued our interest, as reactive astrocytes have been reported to facilitate tumor progression in various cancers [17, 22]. Additionally, the close proximity of GABA and astrocytes led us to speculate whether NSCLC could “tame” astrocytes by secreting GABA (Fig. 6c).

To investigate this further, we co-cultured brain metastatic cells with human astrocytes. The results indicated that control cells attracted astrocytes more effectively than brain metastatic cells that overexpress ABAT. Furthermore, exogenous GABA promoted astrocyte migration to some extent (Fig. 6d-e). We also observed that human astrocytes could enhance the proliferation of tumor cells, with this effect being more pronounced in low-nutrient environments (Fig. 6f-g). This suggests that the close interaction between astrocytes and tumor cells is a critical factor influencing their biological characteristics in metastasis.

Next, we treated astrocytes with GABA and performed qPCR analysis. The results showed that GABA treatment activated inflammasome-related genes in astrocytes, consistent with previous studies linking inflammasome activation to tumor metastasis (Fig. 6h-i). The effect of the NLRP3 inflammasome on the proliferative capacity of metastatic cells was also investigated. The activation of the inflammasome in astrocytes significantly enhanced the proliferation of brain metastatic cells. In contrast, the MCC950-treated group exhibited reduced proliferative capacity (Fig. 6j-k). These findings suggest a positive feedback loop between astrocytes and lung cancer brain metastatic cells, where their interaction within the microenvironment of brain metastases promotes the progression of brain metastasis.

### ABAT expression level is associated with poor survival in NSCLC

**Fig. 7:**
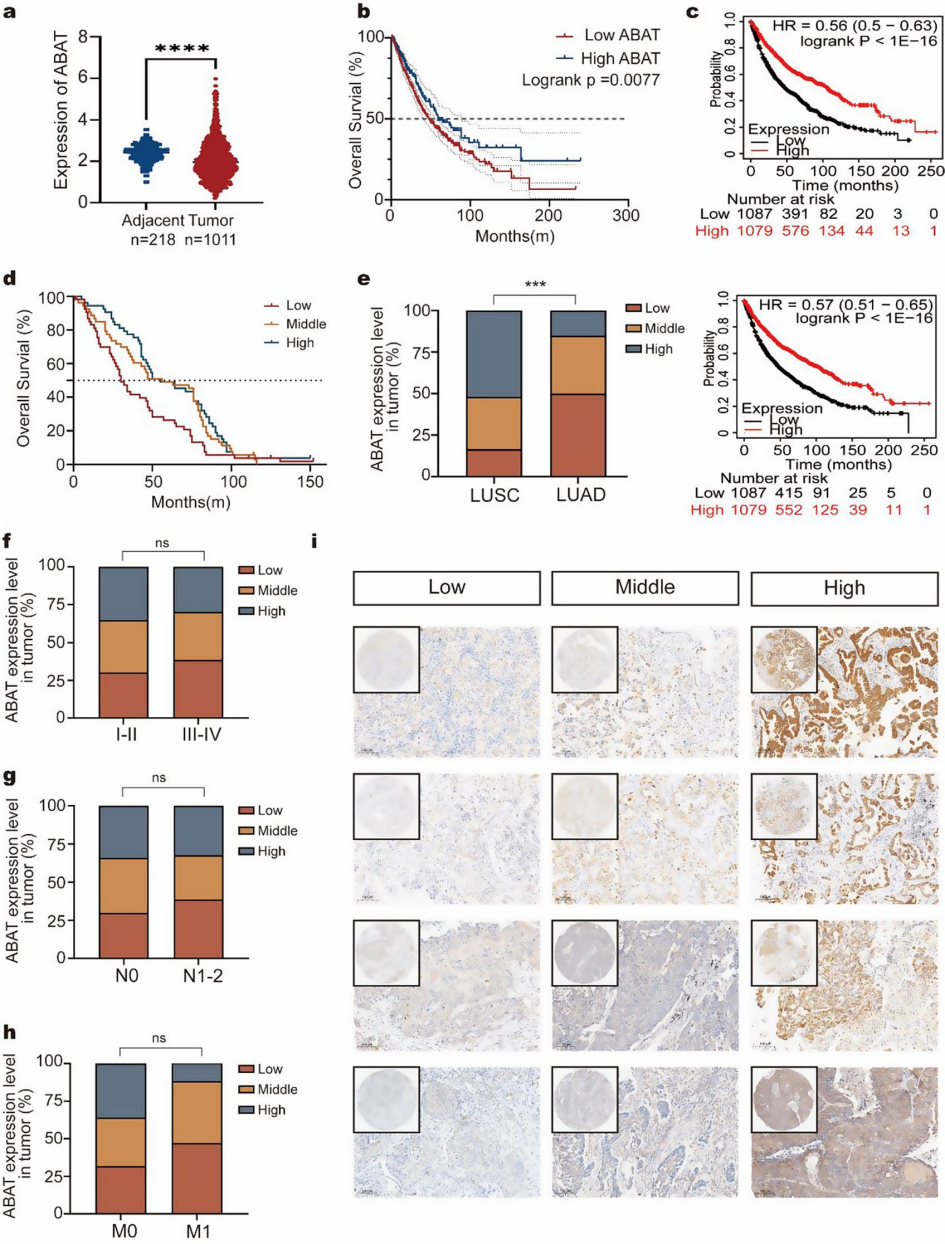
ABAT expression level is associated with poor survival in NSCLC (**a**-**b**) Analysis of ABAT expression in lung cancer and adjacent tissues, along with the correlation between ABAT levels and prognosis using the TCGA database. (**c**) Overall survival curves stratified by ABAT expression levels (ABAT 209459_s_at and 209460_at). (**d**) Kaplan–Meier survival curve analysis was performed to examine the effects of the ABAT on the survival rate in NSCLC (*n* = 159, 80 for LUAD and 79 for LUSC). (**e**) Correlation of ABAT expression with pathological subtype. (**f**) Correlation of ABAT expression with tumor grade. (**g**) Correlation of ABAT expression with lymph node metastasis. (**h**) Correlation of ABAT expression with metastasis. (**i**) Representative images from human NSCLC tumors stained with ABAT. ****p < 0.0001; LUAD, lung adenocarcinoma; LUSC, lung squamous cell carcinoma

We further analyzed ABAT expression using data from the TCGA, which showed that ABAT levels were lower in tumor tissues compared to adjacent tissues (Fig. 7a). Additionally, Kaplan-Meier analysis of TCGA and microarray datasets (209459_s_at and 209460_at) indicated that NSCLC patients with low ABAT levels had poorer survival prognoses, aligning with our findings (Fig. 7b-c). We also examined the expression of ABAT and FOXA2 across various cancer types using the TCGA database. The results revealed that ABAT levels were lower in NSCLC compared to adjacent tissues, with FOXA2 exhibiting a similar trend (Fig. S7a-b). Comparable findings were observed in thyroid carcinoma, bladder urothelial carcinoma, and other malignant tumors. Furthermore, a significant positive correlation was found between ABAT and FOXA2 mRNA levels in NSCLC (Fig. S7c-e).

We also constructed a tissue microarray comprising 159 human NSCLC specimens, which was utilized for IHC analysis. Our findings indicated that NSCLC patients with low ABAT levels had a poorer prognosis (Fig. 7d). The LASSO regression model incorporating ABAT and FOXA2 expression using TCGA datasets also demonstrated similar results (Fig. S8a-d). ABAT expression is also associated with the pathological types of lung cancer, exhibiting higher levels in lung squamous cell carcinoma compared to lung adenocarcinoma (Fig. 7e). Furthermore, the analysis revealed that ABAT expression was not correlated with tumor stage, lymph node metastasis status, or distant metastasis status (Fig. 7f-h). We presented representative IHC images for different ABAT expression groups (Fig. 7i).

## Discussion

In this study, we hypothesize that GABA plays a unique role in the process of NSCLC brain metastasis and the malignant brain tumor microenvironment. To validate this hypothesis, we employed a multi-omics approach combined with in vivo and in vitro experiments. Our findings elucidate the comprehensive mechanisms by which GABA promotes NSCLC brain metastasis and assess its potential as a target for the treatment of NSCLC with brain metastasis.

Brain metastases can lead to severe neurological disorders, cognitive impairment, and emotional difficulties [23]. Previous studies have focused on the role of BBB [24, 25], epithelial-mesenchymal transition [26] and other processes [27–29] in brain metastasis. Metabolic reprogramming is a hallmark event of cancer cells, and the precise molecular mechanism by which metastatic cancer cells undergo metabolic reprogramming to overcome the limitations of the metastatic microenvironment and survive remains unknown. Parida et al. investigated the role of fatty acid metabolism reprogramming in brain metastasis. They discovered that depleting dynamin-related protein 1 in brain metastatic cells limits mitochondrial plasticity. This depletion results in increased lipid droplet accumulation, impaired fatty acid oxidation, and reduced brain metastasis [30]. Another study focused on retinoic acid receptor responder 2 (RARRES2), an important adipokine that regulates lipogenesis and adipocyte lipid metabolism. The results demonstrated that RARRES2 mediates lipid metabolism reprogramming by modulating the PTEN-mTOR-SREBP1 signaling pathway, leading to increased glycerophospholipid levels and decreased triglyceride levels and promoting the proliferation of breast cancer cells within the brain microenvironment [31]. We focus on GABA, a traditional inhibitory neurotransmitter [32], highlighting the elevated GABA secretion by brain metastasis NSCLC cells and its association with high expression levels in patients with brain metastases from NSCLC. This means that GABA may play an important role in the pathological process of brain metastasis. Specifically, we found that tumor cells with brain metastasis tendency could inhibit ABAT by inhibiting FOXA2 expression, thereby reducing the breakdown of GABA and increasing the accumulation of GABA. GABA is known to be a non-protein amino acid. Recent studies have begun to focus on its role beyond signal transduction. Studies showed that GABA not only can limit anti-tumor immunity [33, 34], but also can promote the progression of tumors [12], which emphasizes the its tumor-promoting properties.

Through metabolome and transcriptome sequencing, we found that lung cancer cells prone to brain metastasis exhibit high expression of GABAergic-related pathways. At the same time, we found that this characteristic is stably inherited. These findings indicate that lung cancer cells undergoing brain metastasis possess a unique reprogramming ability, altering their original characteristics at the genetic level to enhance their potential for metastasis to the brain. ABAT is a GABA-degrading enzyme that exhibits similar trends in both WB and qPCR analyses of parental and brain metastatic cells. We speculate that ABAT may be a key molecule involved in altering GABA during lung cancer brain metastasis. ABAT was previously considered to be crucial for mitochondrial nucleoside metabolism [35]. As a protein with higher expression in para-cancer than in tumors, its role in tumor development has been less explored [36–38]. In our analysis, we found that ABAT expression varied according to the pathological type of lung cancer. Specifically, the expression level was higher in squamous cell carcinoma than in adenocarcinoma. Furthermore, the loss of ABAT was associated with increased malignancy in NSCLC, whereas its overexpression inhibited tumor progression. Additionally, we discovered that the suppression of tumor malignancy caused by ABAT overexpression could be further enhanced by exogenous GABA to some extent. This finding aligns with ABAT’s role in decomposing GABA and underscores the significant role of GABA in the process of brain metastasis. GABA was previously thought to serve as a carbon source to provide energy for the tricarboxylic acid cycle. However, in our study, we did not observe changes in ATP in tumor cells after GABA treatment in this study, which is somewhat similar with the study of De Huang et. al [34]. The disruption of GABA metabolism caused by the absence of ABAT may be the reason affecting its entry into the TCA cycle. Activation of the NF-κB signaling pathway is considered to be a dominant process related to carcinogenesis [19, 39, 40]. Our data indicated that this pathway is enriched in tumor cells after GABA treatment and the inhibitors can effectively reduce the malignant phenotype of tumors. In total, in our study, we confirmed the role of ABAT/GABA axis in lung cancer brain metastasis and emphasis its ability to activate the NF-κB signaling pathway.

Previous studies have also suggested that immune cells in the tumor microenvironment play different roles in promoting or suppressing cancer at different stages of metastasis [41]. Our study revealed that astrocytes frequently form a distinct physical barrier at the periphery of brain metastases, highlighting their essential role in the brain metastasis process. As the main stromal cells in the brain, astrocytes form a physical barrier that prevents immune cells from clearing brain metastases, which was similar to the previously reported function of fibroblasts [42, 43]. At the same time, accumulating evidence indicates that tumor cells can tame the astrocyte to promote its colonization in the CNS microenvironment [44]. In our study, we found that tumor cells can mimic neurons, the main source of GABA in the brain, by expressing high levels of GABA, thereby promoting inflammasome activation of astrocytes and creating a pro-cancer microenvironment, which was also found in breast cancer [45]. The crosstalk between tumor and astrocytes is mediated by GABA, which gives GABA a new identity in lung cancer brain metastasis and also suggests the importance of targeting GABA.

**Fig. 8:**
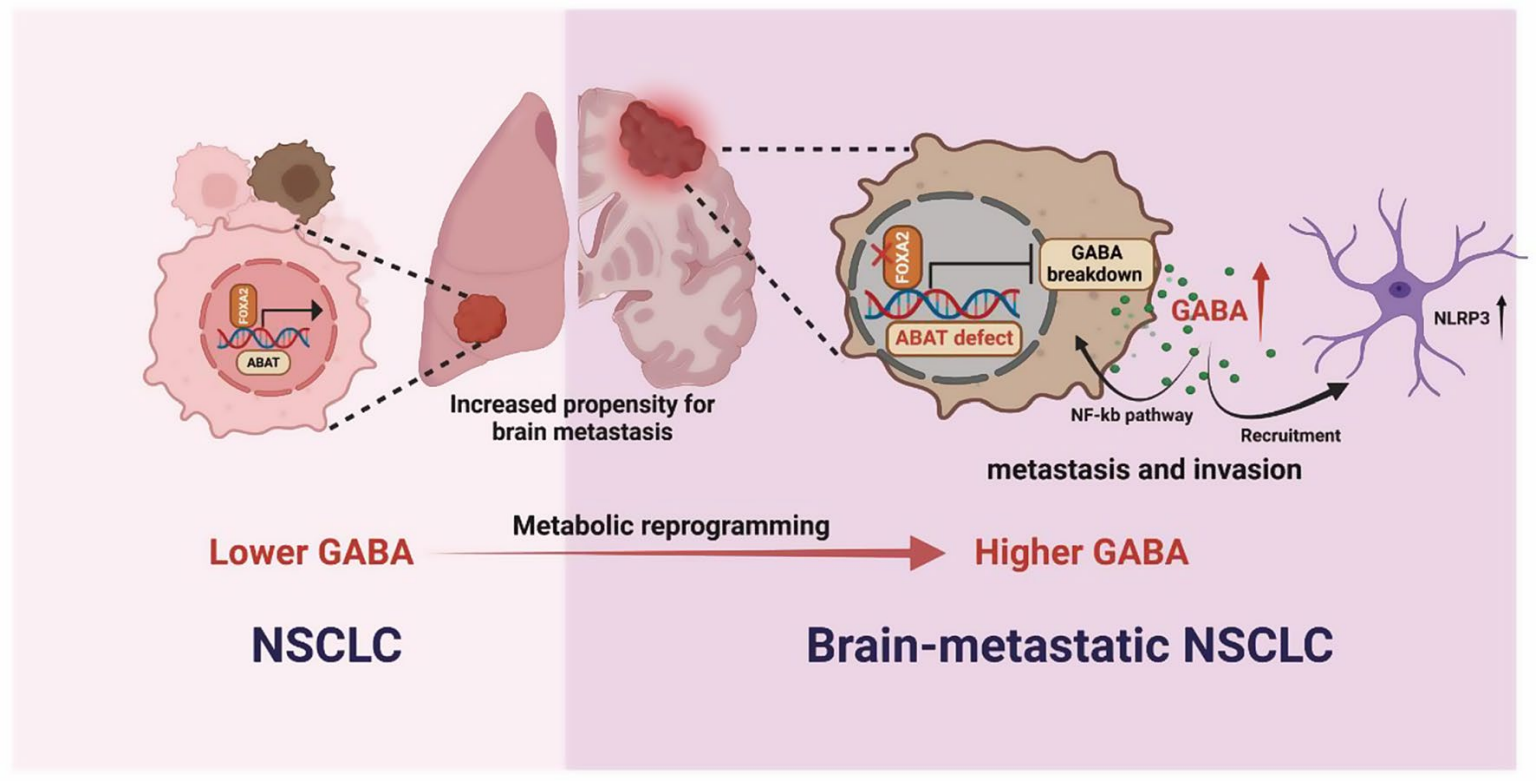
Schematic model illustrating the mechanism of GABA-mediated brain metastasis in NSCLC

## Conclusions

In summary, through transcriptome and metabolome analyses of parental and brain metastatic cells, we identified and explored the potential mechanisms by which GABA promotes brain metastasis in NSCLC. Our findings indicate that lung cancer cells can activate the NF-κB pathway via the FOXA2/ABAT/GABA axis, thereby facilitating brain metastasis. The interaction between NSCLC and astrocytes also constructs an inhibitory microenvironment, and promote colonization. This study provides a theoretical foundation for considering GABA as a potential therapeutic target for patients with brain metastasis (Fig. 8).

## Electronic supplementary material

Below is the link to the electronic supplementary material.

Supplementary Material 1

Supplementary Material 2

## Data Availability

No datasets were generated or analysed during the current study.

## Abbreviations

NSCLC: Non-small cell lung cancer
GABA: γ-Aminobutyric acid
CNS: Central nervous system
ABAT: 4-aminobutyrate aminotransferase
FOXA2: Forkhead box A2
Luc: Luciferase
GFP: Green fluorescent protein
HA1800: Human astrocyte
BrM: Brain metastasis
CCK 8: Cell counting kit 8
EdU: 5-ethynyl-2′-deoxyuridine
PBS: Phosphate-buffered saline
OE: Over expression
NC: Negative control
LC: MS-Liquid chromatography-mass spectrometry
HE: Hematoxylin-eosin
PPP: Photons per pixel
FFEP: Formalin-fixed paraffin-embedded
qPCR: Quantitative real-time polymerase chain reaction
WB: Western blot
ELISA: Enzyme linked immunosorbent assay
ATP: Adenosine triphosphate
IHC: Immunohistochemical
mIF: Multiplex immunofluorescence
BBB: Blood-brain barrier
TCA: Tricarboxylic acid cycle
TME: Tumor microenvironment
RARRES2: Retinoic acid receptor responder 2
