# Supplementary material for: GABA regulates metabolic reprogramming to mediate the development of brain metastasis in non-small cell lung cancer
Source: J Exp Clin Cancer Res. 2025 Feb 19;44:61. doi: 10.1186/s13046-025-03315-9 (PMC11837350; doi:10.1186/s13046-025-03315-9)
Supplement: Supplementary file 1 — Supplementary Material 1 [file 13046_2025_3315_MOESM1_ESM.docx]

**Supplementary Figure 1. Brain-metastatic NSCLC presented with higher proliferation.**

**
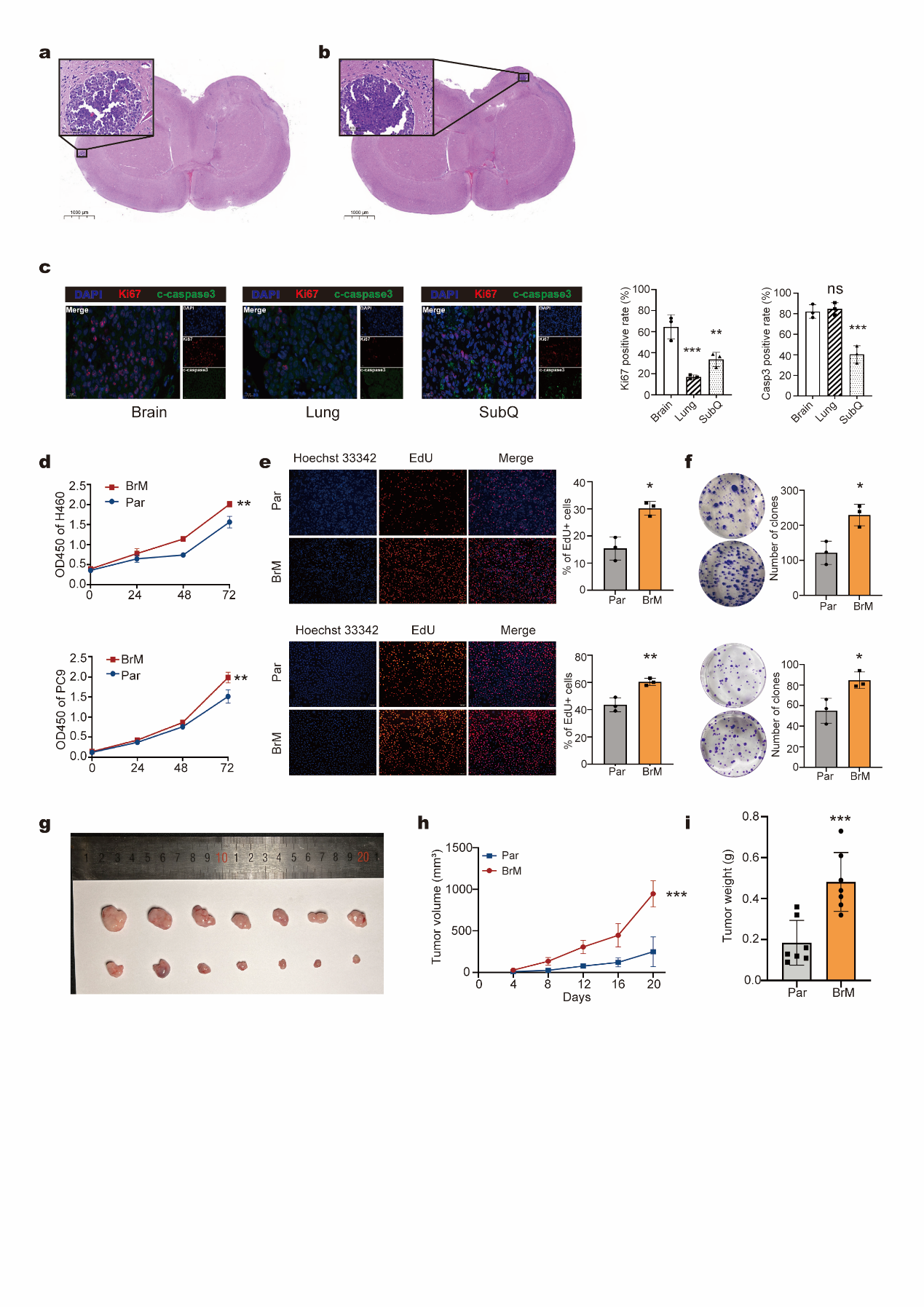
**

**(a-b)** Representative images of HE staining illustrating NSCLC with brain metastasis in mice. **(c)** Representative images and quantification of immunofluorescence staining for Ki67 and cleaved caspase-3 in brain, lung, and subcutaneous tumors (SubQ). **(d-f)** CCK-8 assays, EdU assays, and colony formation assays were conducted to assess cell viability between parental cells (H460 and PC9) and brain-metastatic cells (H460BrM and PC9BrM). **(g-i)** H460 or H460BrM cells were implanted subcutaneously into BALB/c nude mice. Tumor volumes were measured every four days post-injection. Approximately three weeks later, all tumors were excised and photographed, with tumor growth curves and weights presented.

*p<0.05; **p<0.01; ***p<0.001; ns, not significant. NSCLC, non-small cell lung cancer. Par, Parental cells; BrM, Brain metastasis cells.

**Supplementary Figure 2. Brain-metastatic NSCLC presented with higher capability of metastasis.**

**
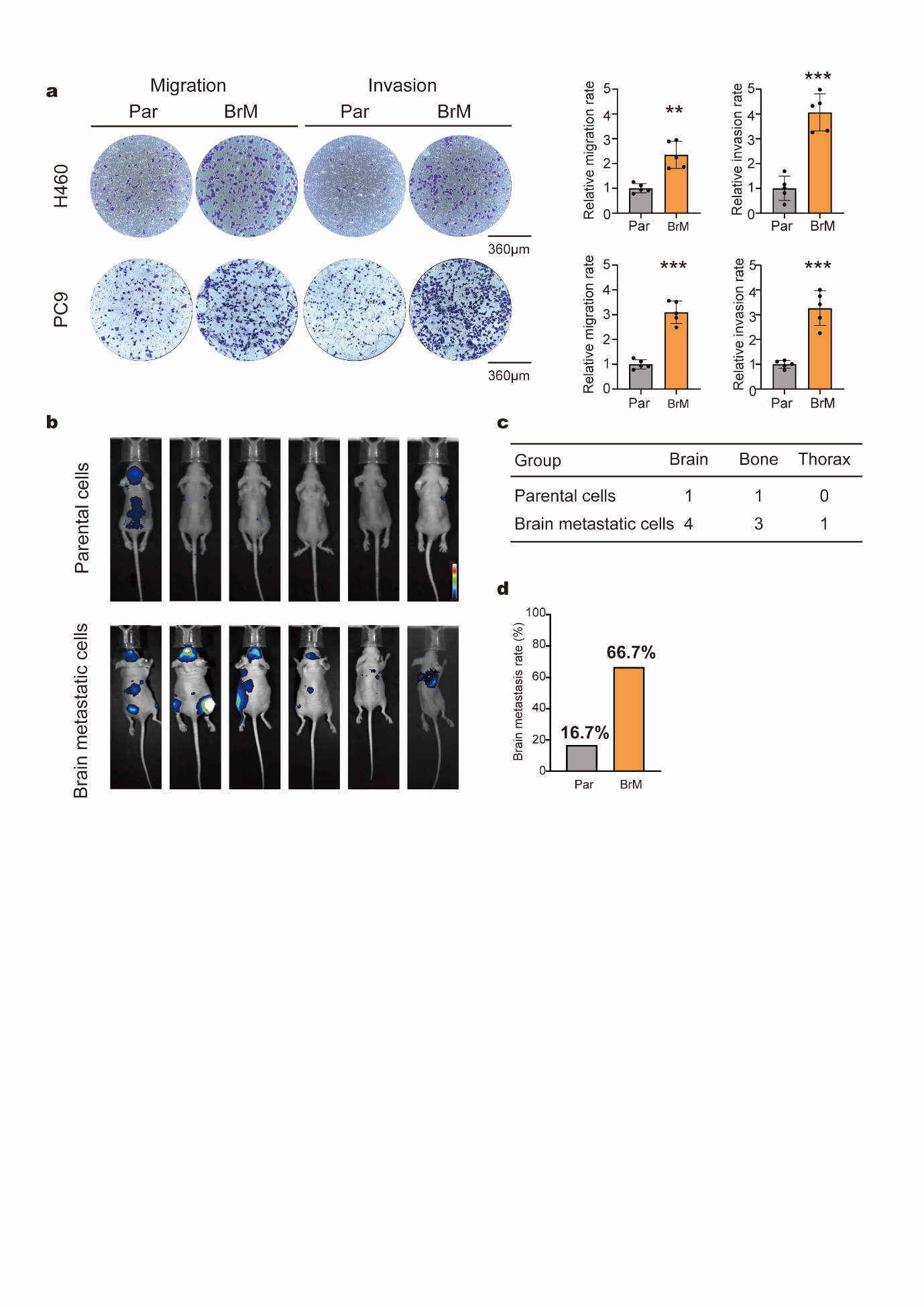
**

**(a)** Transwell assays were employed to assess the migration and invasion capabilities of parental versus brain-metastatic cells. **(b-d)** In vivo imaging was performed to illustrate and compare the brain metastasis rates following left ventricular injection of H460 and H460BrM cells.

**p<0.01; ***p<0.001. NSCLC, non-small cell lung cancer. Par, Parental cells; BrM, Brain metastasis cells.

**Supplementary Figure 3. GABA promote the brain metastasis of NSCLC in vivo**

**
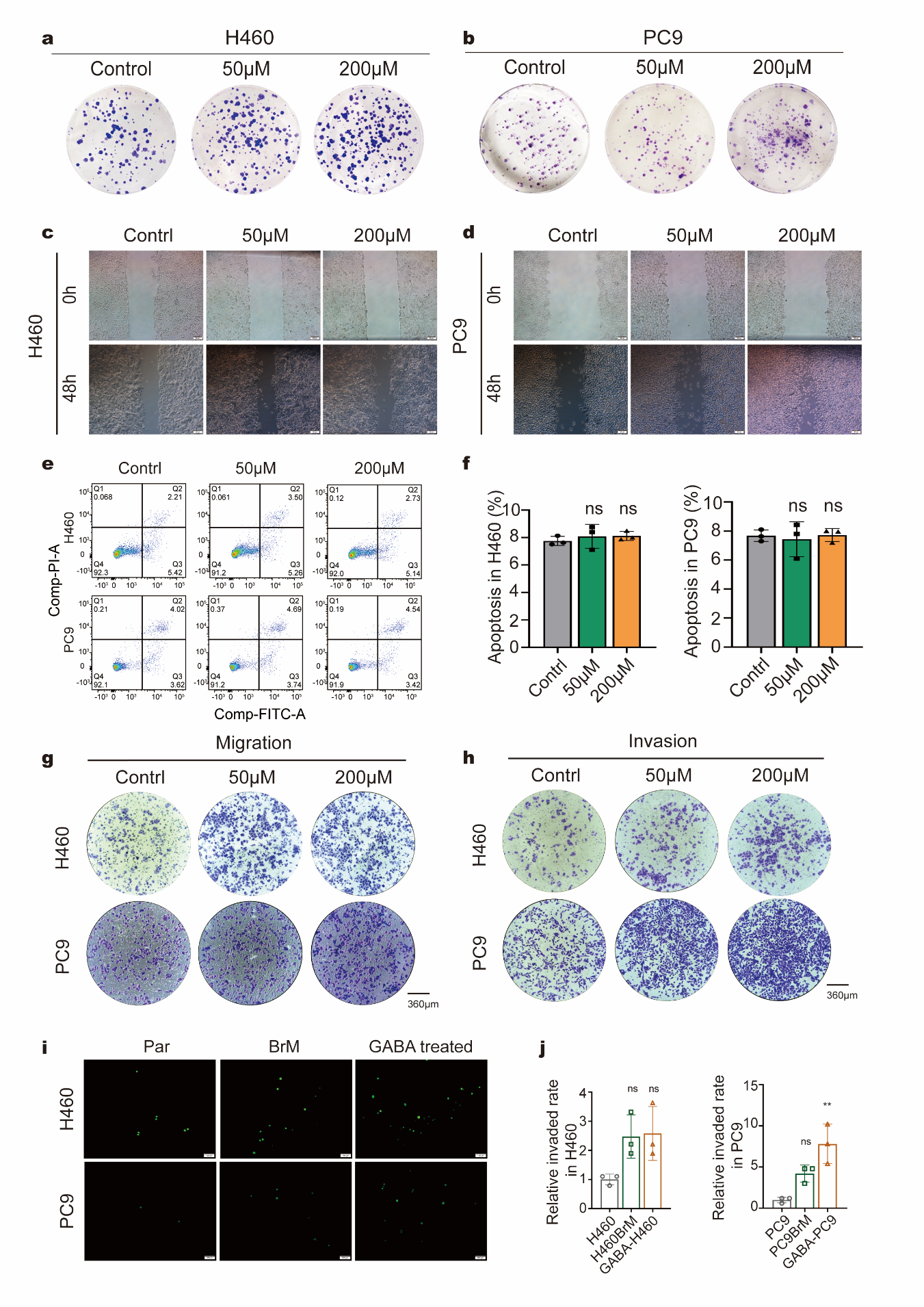
**

**(a-b)** Representative images of colony formation by H460 and PC9 cells after treatment with GABA (0 μM, 50 μM, and 200 μM). **(c-d)** Representative images of wound healing assays by H460 and PC9 cells after treatment with GABA. **(e-f)** The apoptotic rates of H460 and PC9 cells treated with GABA were analyzed using flow cytometry. **(g-h)** Transwell assays were conducted to compare the migration and invasion capabilities of H460 and PC9 cells treated with different concentrations of GABA. **(i-j)** Representative images and statistical analysis of the ability of parental cells, brain metastatic cells, and GABA-treated cells to cross the BBB.

**p<0.01; ns, not significant. Par, Parental cells; BrM, Brain metastasis cells. BBB, blood-brain barrier.

**Supplementary Figure 4. qPCR comparison of NF-κB signaling pathway-related genes between parental cells and brain metastatic cells.**

**
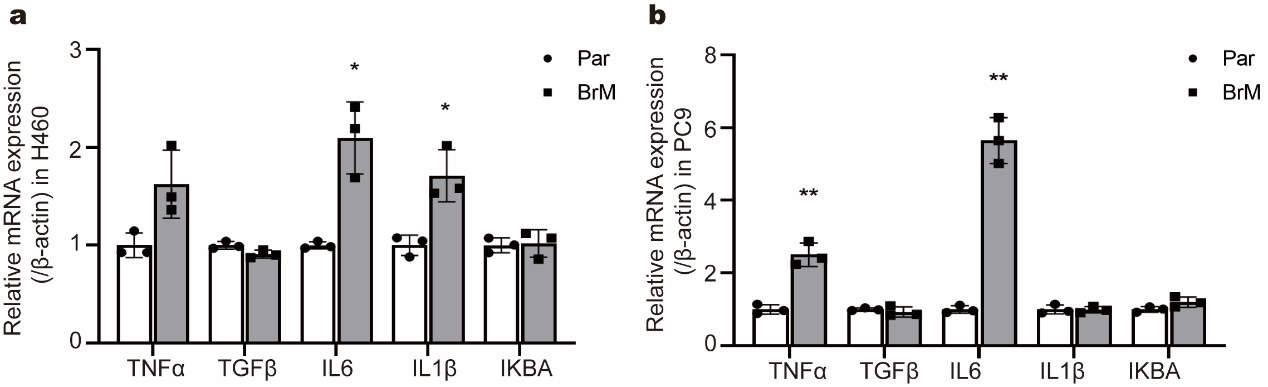
**

**(a**) qPCR comparison of NF-κB signaling pathway-related genes between parental cells and brain metastatic cells in H460. **(b)** qPCR comparison of NF-κB signaling pathway-related genes between parental cells and brain metastatic cells in PC9.

*p<0.05; **p<0.01. Par, Parental cells; BrM, Brain metastasis cells.

**Supplementary Figure 5. Loss of ABAT promote the malignancy of NSCLC.**

**
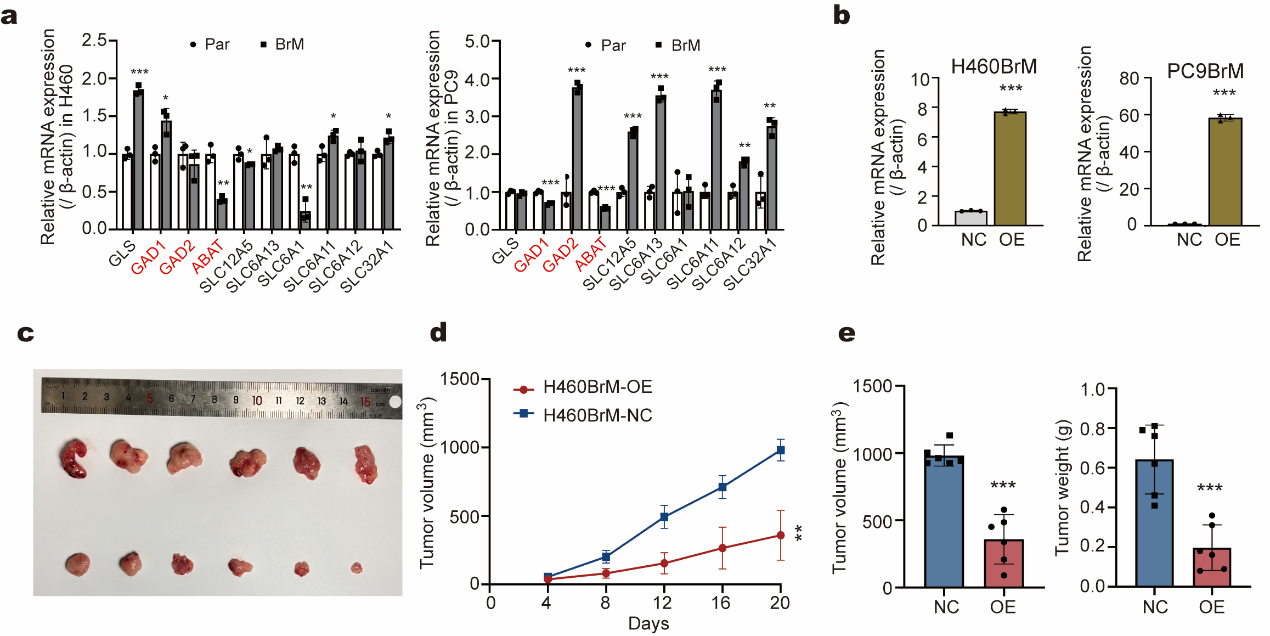
**

**(a)** qPCR analysis showing changes in GABA-related gene expression in brain-metastatic cells (H460BrM and PC9BrM) compared to parental (H460 and PC9). **(b)** Confirmation of ABAT overexpression at the mRNA level in H460BrM and PC9BrM cells. **(c-e)** H460BrM-NC or H460BrM-OE cells were implanted subcutaneously into BALB/c nude mice. Approximately three weeks later, all tumors were photographed. The results include tumor growth curves, tumor volume, and tumor weights.

*p<0.05; **p<0.01; ***p<0.001. Par, Parental cells; BrM, Brain metastasis cells. GAD1, Glutamate decarboxylase 1; GAD2, Glutamate decarboxylase 2; ABAT, 4-aminobutyrate aminotransferase.

**Supplementary Figure 6. Knockdown of FOXA2 enhances the malignancy of brain-metastatic NSCLC with ABAT overexpression**

**
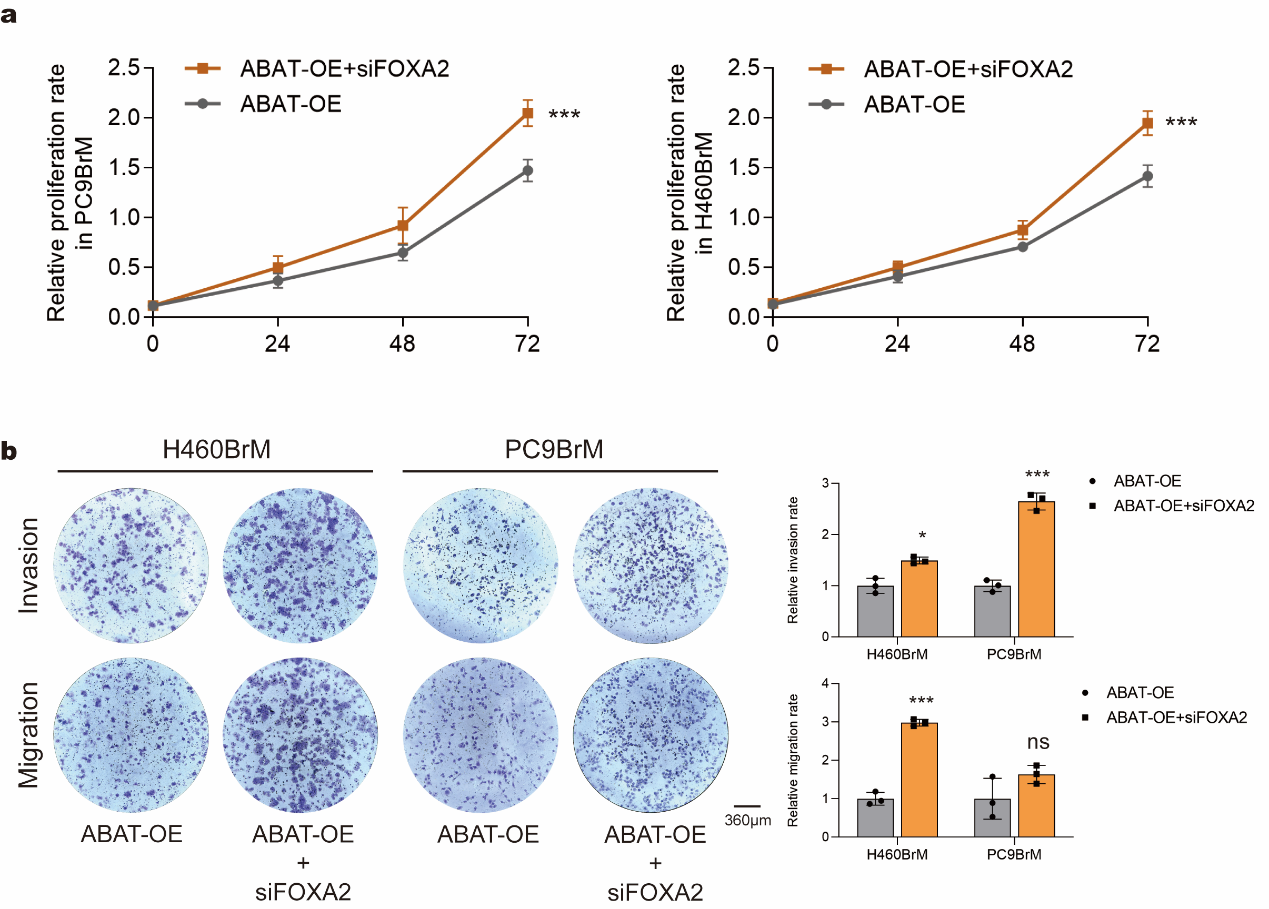
**

**(a)** CCK-8 assays were conducted to compare the proliferation rates of brain metastatic cells overexpressing ABAT, with and without FOXA2 knockdown. **(b)** Transwell assays were employed to assess the migration and invasion capabilities of brain metastatic cells overexpressing ABAT, with and without FOXA2 knockdown.

*p<0.05; ***p<0.001; ns, not significant. NSCLC, non-small cell lung cancer. BrM, Brain metastasis cells. OE, overexpression.

**Supplementary Figure 7. Expression of ABAT and FOXA2 across various cancer types and their correlation in NSCLC**

**
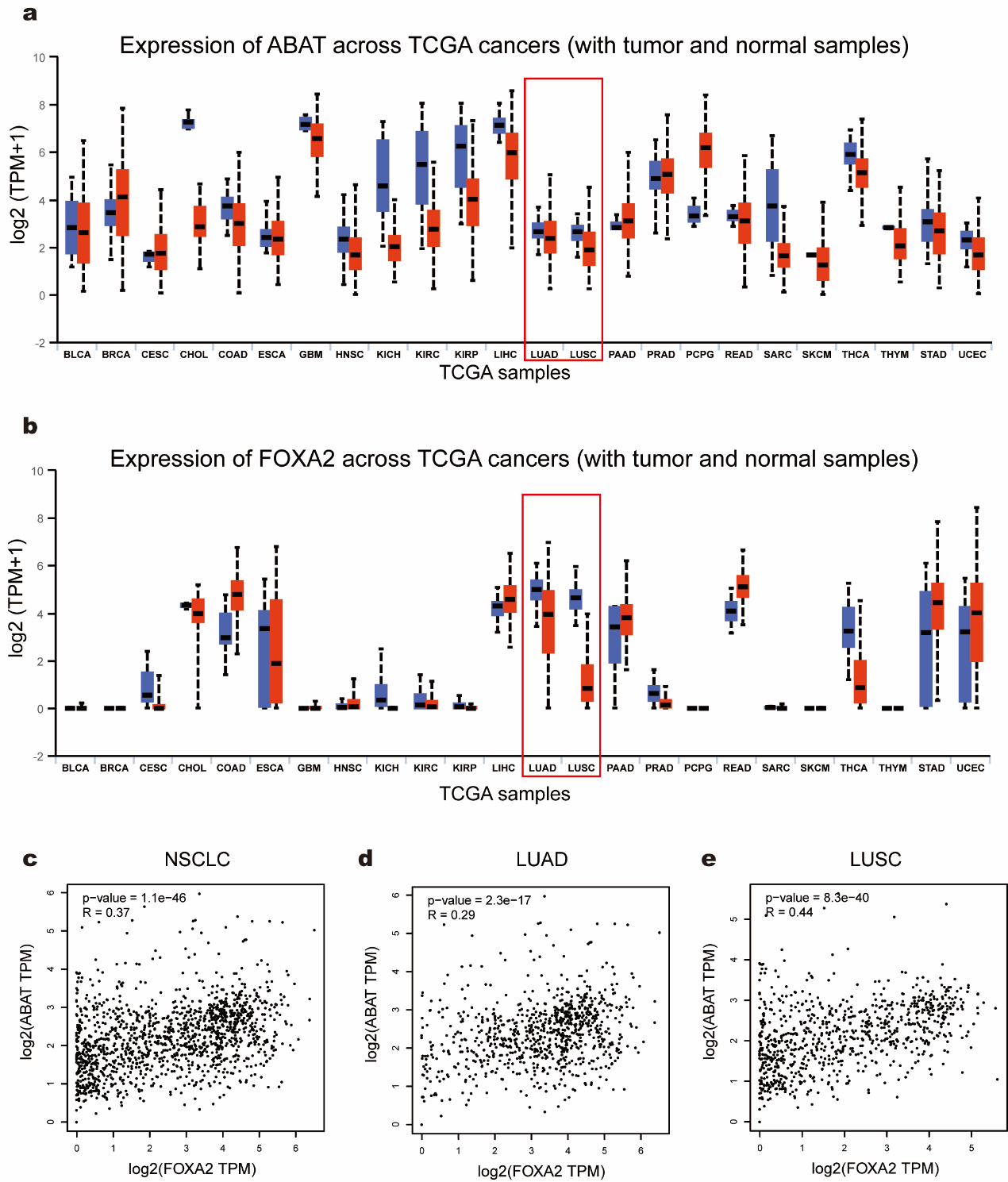
**

**(a)** Analyzing the expression of ABAT in tumors (red) and adjacent normal tissues (blue) across various cancer types using the TCGA database. **(b)** Analyzing the expression of FOXA2 in tumors (red) and adjacent normal tissues (blue) across various cancer types using the TCGA database. **(c-e)** Analyze the correlation between ABAT and FOXA2 in NSCLC, LUAD and LUSC utilizing the TCGA database.

**Supplementary Figure 8.** **Establishment and validation of RiskScore**

**
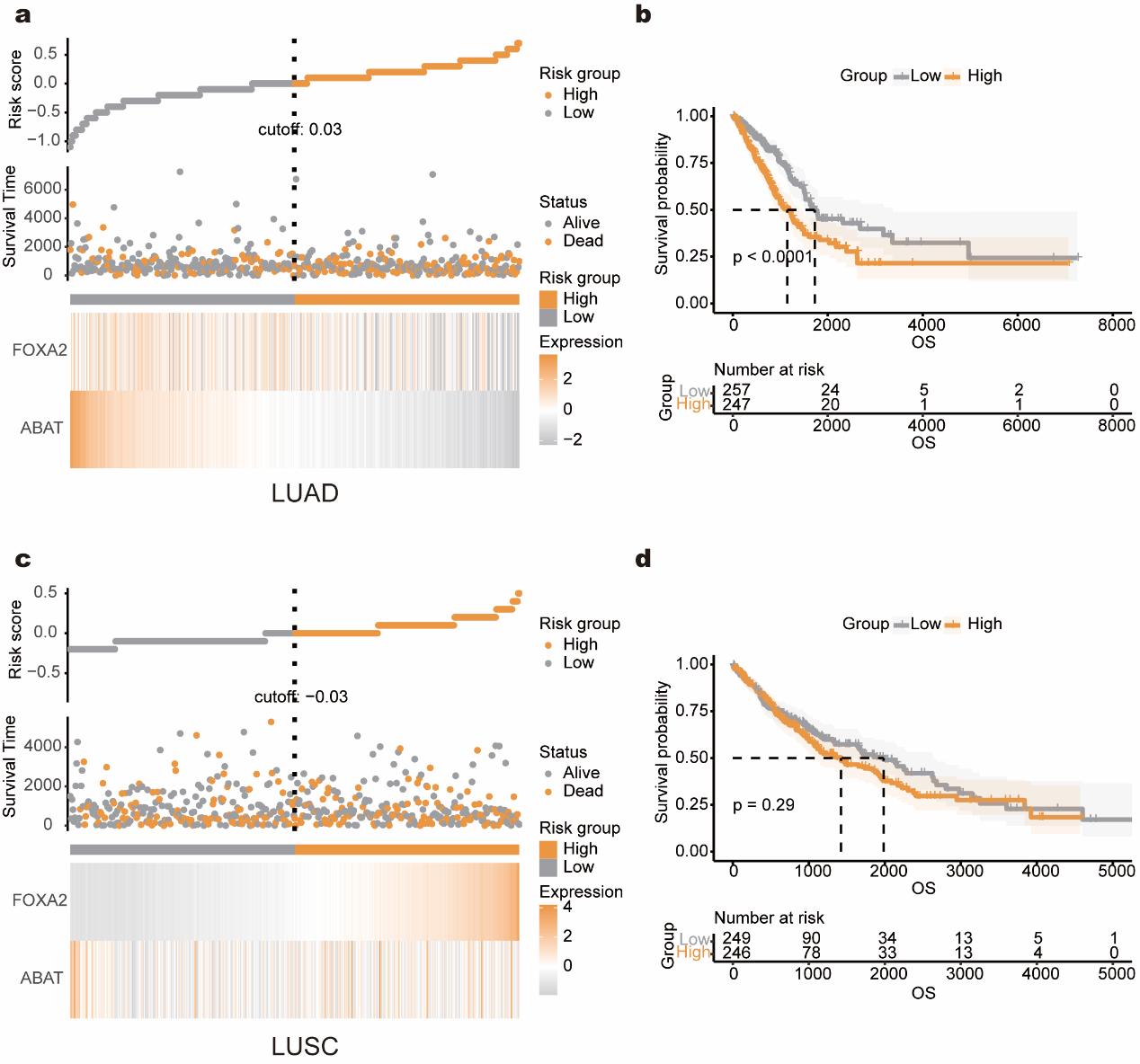
**

**(a, c)** Risk score, survival time, status, and gene expression of LUAD and LUSC in the TCGA dataset. **(b, d)** KM survival curves of high and low RiskScore groups of LUAD and LUSC in the TCGA dataset.
