# Supplementary material for: GABA regulates metabolic reprogramming to mediate the development of brain metastasis in non-small cell lung cancer
Source: J Exp Clin Cancer Res. 2025 Feb 19;44:61. doi: 10.1186/s13046-025-03315-9 (PMC11837350; doi:10.1186/s13046-025-03315-9)
Supplement: Supplementary file 2 — Supplementary Material 2 [file 13046_2025_3315_MOESM2_ESM.docx]

**Supplementary Table 1. The sequences of primers and siRNA**

| **Gene** | | **Sequence (5’-3’)** |
| --- | --- | --- |
| **qRT-PCR primers** | | |
| *TNFα* | Forward primer | GAGGACCTGGGAGTAGATGAG |
|  | Reverse primer | CCTCTCTCTAATCAGCCCTCTG |
| *TGFβ* | Forward primer | GTGGGTTTCCACCATTAGCAC |
|  | Reverse primer | GGCCAGATCCTGTCCAAGC |
| *IL6* | Forward primer | CCATCTTTGGAAGGTTCAGGTTG |
|  | Reverse primer | ACTCACCTCTTCAGAACGAATTG |
| *IL1β* | Forward primer | GTCGGAGATTCGTAGCTGGA |
|  | Reverse primer | ATGATGGCTTATTACAGTGGCAA |
| *IKBA* | Forward primer | GCCATTGTAGTTGGTAGCCTTCA |
|  | Reverse primer | CTCCGAGACTTTCGAGGAAATAC |
| *GLS* | Forward primer | TCTACAGGATTGCGAACGTCT |
|  | Reverse primer | CTTTGTCTAGCATGACACCATCT |
| *GAD1* | Forward primer | GCGGACCCCAATACCACTAAC |
|  | Reverse primer | CACAAGGCGACTCTTCTCTTC |
| *GAD2* | Forward primer | TTTTGGTCTTTCGGGTCGGAA |
|  | Reverse primer | TTCTCGGCGTCTCCGTAGAG |
| *ABAT* | Forward primer | AAGAGAGCCGAGGCAATTACC |
|  | Reverse primer | GCTCGCATTTTGAGGCTGTTG |
| *SLC12A5* | Forward primer | AGGAAAGCAGTCCCTTCATCA |
|  | Reverse primer | GCCTCTTCATGCTCCCTACTT |
| *SLC6A13* | Forward primer | TCTCAGGCACAACCAGTAATGG |
|  | Reverse primer | CCTAAGCCAATGATCTCCCCA |
| *SLC6A1* | Forward primer | GGGTATGGAAGCTGGCTCCTA |
|  | Reverse primer | AGGGGTTGTCGCACTGTTTC |
| *SLC6A11* | Forward primer | CTGATTCCCTACGTGGTGTTTT |
|  | Reverse primer | CACCTGTGTTGCATAGCCAAT |
| *SLC6A12* | Forward primer | CGGAGGTGGAGCCTTCTTC |
|  | Reverse primer | CTGGTGTATTGGCCCAACG |
| *SLC32A1* | Forward primer | ACGTCCGTGTCCAACAAGTC |
|  | Reverse primer | AAAGTCGAGGTCGTCGCAATG |
| *NFIC* | Forward primer | GCTGGACACGACCGACTTC |
|  | Reverse primer | CCGGGACACTTGGATGAGC |
| *TFAP2C* | Forward primer | TCAGTCCCTGGAAGATTGTCG |
|  | Reverse primer | CCAGTAACGAGGCATTTAAGCA |
| *TP63* | Forward primer | GGACCAGCAGATTCAGAACGG |
|  | Reverse primer | AGGACACGTCGAAACTGTGC |
| *CREB1* | Forward primer | ATTCACAGGAGTCAGTGGATAGT |
|  | Reverse primer | CACCGTTACAGTGGTGATGG |
| *GATA2* | Forward primer | ACTGACGGAGAGCATGAAGAT |
|  | Reverse primer | CCGGCACATAGGAGGGGTA |
| *MYC* | Forward primer | TCCCTCCACTCGGAAGGAC |
|  | Reverse primer | CTGGTGCATTTTCGGTTGTTG |
| *FOXA2* | Forward primer | GGAGCAGCTACTATGCAGAGC |
|  | Reverse primer | CGTGTTCATGCCGTTCATCC |
| *NLRP3* | Forward primer | GATCTTCGCTGCGATCAACAG |
|  | Reverse primer | CGTGCATTATCTGAACCCCAC |
| *P2RX7* | Forward primer | TATGAGACGAACAAAGTCACTCG |
|  | Reverse primer | GCAAAGCAAACGTAGGAAAAGAT |
| *CASP1* | Forward primer | TTTCCGCAAGGTTCGATTTTCA |
|  | Reverse primer | GGCATCTGCGCTCTACCATC |
| *IL1B* | Forward primer | ATGATGGCTTATTACAGTGGCAA |
|  | Reverse primer | GTCGGAGATTCGTAGCTGGA |
| *IL1A* | Forward primer | TGGTAGTAGCAACCAACGGGA |
|  | Reverse primer | ACTTTGATTGAGGGCGTCATTC |
| *IL18* | Forward primer | TCTTCATTGACCAAGGAAATCGG |
|  | Reverse primer | TCCGGGGTGCATTATCTCTAC |
| *IL1R2* | Forward primer | ATGTTGCGCTTGTACGTGTTG |
|  | Reverse primer | CCCGCTTGTAATGCCTCCC |
| *β-ACTIN* | Forward primer | CTCCTTAATGTCACGCACGAT |
|  | Reverse primer | CATGTACGTTGCTATCCAGGC |
| *Nlrp3 (mouse)* | Forward primer | ATTACCCGCCCGAGAAAGG |
|  | Reverse primer | CATGAGTGTGGCTAGATCCAAG |
| *P2rx7 (mouse)* | Forward primer | CTGGAACGATGTCTTGCAGTAT |
|  | Reverse primer | CCCACTTGACGGTGCCATAAT |
| *Casp1 (mouse)* | Forward primer | AATACAACCACTCGTACACGTC |
|  | Reverse primer | AGCTCCAACCCTCGGAGAAA |
| *Il1b (mouse)* | Forward primer | GAAATGCCACCTTTTGACAGTG |
|  | Reverse primer | TGGATGCTCTCATCAGGACAG |
| *Il1a (mouse)* | Forward primer | CGAAGACTACAGTTCTGCCATT |
|  | Reverse primer | GACGTTTCAGAGGTTCTCAGAG |
| *Il18 (mouse)* | Forward primer | GACTCTTGCGTCAACTTCAAGG |
|  | Reverse primer | CAGGCTGTCTTTTGTCAACGA |
| *Il1r2 (mouse)* | Forward primer | GTTTCTGCTTTCACCACTCCA |
|  | Reverse primer | GAGTCCAATTTACTCCAGGTCAG |
| *Actb (mouse)* | Forward primer | GTGACGTTGACATCCGTAAAGA |
|  | Reverse primer | GCCGGACTCATCGTACTCC |
| **Sequences for siRNA** | | |
| FOXA2 #1 | Sense sequence | CCAUGAACAUGUCGUCGUATT |
|  | Antisense sequence | UACGACGACAUGUUCAUGGTT |
| FOXA2 #2 | Sense sequence | CGGGCAACAUGUUCGAGAATT |
|  | Antisense sequence | UUCUCGAACAUGUUGCCCGTT |
